# RNAi-mediated depletion of the NSL complex subunits leads to abnormal chromosome segregation and defective centrosome duplication in *Drosophila* mitosis

**DOI:** 10.1371/journal.pgen.1008371

**Published:** 2019-09-17

**Authors:** Gera A. Pavlova, Julia V. Popova, Evgeniya N. Andreyeva, Lyubov A. Yarinich, Mikhail O. Lebedev, Alyona V. Razuvaeva, Tatiana D. Dubatolova, Anastasiya L. Oshchepkova, Claudia Pellacani, Maria Patrizia Somma, Alexey V. Pindyurin, Maurizio Gatti

**Affiliations:** 1 Institute of Molecular and Cellular Biology, Siberian Branch of RAS, Novosibirsk, Russia; 2 Institute of Cytology and Genetics, Siberian Branch of RAS, Novosibirsk, Russia; 3 Novosibirsk State University, Novosibirsk, Russia; 4 Institute of Chemical Biology and Fundamental Medicine, Siberian Branch of RAS, Novosibirsk, Russia; 5 IBPM CNR c/o Department of Biology and Biotechnology, Sapienza University of Rome, Rome, Italy; Geisel School of Medicine at Dartmouth, UNITED STATES

## Abstract

The *Drosophila* Nonspecific Lethal (NSL) complex is a major transcriptional regulator of housekeeping genes. It contains at least seven subunits that are conserved in the human KANSL complex: Nsl1/Wah (KANSL1), Dgt1/Nsl2 (KANSL2), Rcd1/Nsl3 (KANSL3), Rcd5 (MCRS1), MBD-R2 (PHF20), Wds (WDR5) and Mof (MOF/KAT8). Previous studies have shown that Dgt1, Rcd1 and Rcd5 are implicated in centrosome maintenance. Here, we analyzed the mitotic phenotypes caused by RNAi-mediated depletion of Rcd1, Rcd5, MBD-R2 or Wds in greater detail. Depletion of any of these proteins in *Drosophila* S2 cells led to defects in chromosome segregation. Consistent with these findings, *Rcd1*, *Rcd5* and *MBD-R2* RNAi cells showed reduced levels of both Cid/CENP-A and the kinetochore component Ndc80. In addition, RNAi against any of the four genes negatively affected centriole duplication. In Wds-depleted cells, the mitotic phenotypes were similar but milder than those observed in Rcd1-, Rcd5- or MBD-R2-deficient cells. RT-qPCR experiments and interrogation of published datasets revealed that transcription of many genes encoding centromere/kinetochore proteins (e.g., *cid*, *Mis12* and *Nnf1b*), or involved in centriole duplication (e.g., *Sas-6*, *Sas-4* and *asl*) is substantially reduced in *Rcd1*, *Rcd5* and *MBD-R2* RNAi cells, and to a lesser extent in *wds* RNAi cells. During mitosis, both Rcd1-GFP and Rcd5-GFP accumulate at the centrosomes and the telophase midbody, MBD-R2-GFP is enriched only at the chromosomes, while Wds-GFP accumulates at the centrosomes, the kinetochores, the midbody, and on a specific chromosome region. Collectively, our results suggest that the mitotic phenotypes caused by Rcd1, Rcd5, MBD-R2 or Wds depletion are primarily due to reduced transcription of genes involved in kinetochore assembly and centriole duplication. The differences in the subcellular localizations of the NSL components may reflect direct mitotic functions that are difficult to detect at the phenotypic level, because they are masked by the transcription-dependent deficiency of kinetochore and centriolar proteins.

## Author summary

The *Drosophila* Nonspecific Lethal (NSL) complex is a conserved protein assembly that controls transcription of more than 4,000 housekeeping genes. We analyzed the mitotic functions of four genes, *Rcd1*, *Rcd5*, *MBD-R2* and *wds*, encoding NSL subunits. Inactivation of these genes by RNA interference (RNAi) resulted in defects in both chromosome segregation and centrosome duplication. Our analyses indicate that RNAi against *Rcd1*, *Rcd5* or *MBD-R2* reduces transcription of genes involved in centromere/kinetochore assembly and centriole replication. During interphase, Rcd1, Rcd5, MBD-R2 and Wds are confined to the nucleus, as expected for transcription factors. However, during mitosis each of these proteins relocates to specific mitotic structures. Our results suggest that the four NSL components work together as a complex to stimulate transcription of genes encoding important mitotic determinants. However, the different localization of the proteins during mitosis suggests that they might have acquired secondary “moonlighting” functions that directly contribute to the mitotic process.

## Introduction

The spindle is a microtubule (MT)-based highly dynamic molecular machine that mediates chromosome segregation during cell division. Spindle formation requires many proteins that specifically bind and/or regulate MT assembly and dynamics, and also some proteins that are associated with the chromatin during interphase [1]. These latter proteins dissociate from the chromosomes upon mitotic entry and return to the nucleus during telophase; they have therefore functional roles in both interphase chromatin and in mitotic spindle assembly. Examples of these proteins are the components of the human KAT8-associated nonspecific lethal (KANSL) complex, which includes at least seven proteins: KANSL1, KANSL2, KANSL3, MCRS1, PHF20, WDR5 and the MOF/KAT8 histone acetyltransferase. The KANSL complex localizes in the nucleus of interphase cells where it regulates transcription of a specific set of genes and contributes to stem cell identity [2, 3]. Mutations in *KANSL1* dominantly induce the Koolen-de Vries syndrome characterized by mental retardation and peculiar facial features, and mutations in *KANSL2* have been associated with intellectual disabilities [4–6]. Studies on human cells and *Xenopus* egg extracts have shown that during mitosis, KANSL1, KANSL3 and MCRS1 re-localize from the chromatin to the MT minus ends of the mitotic spindle, playing essential roles in spindle assembly and chromosome segregation [7, 8]. Also WDR5 associates with the spindle during mitosis, and its RNAi-mediated depletion leads to spindle defects and chromosome misalignment [9]. Thus, the subunits of the KANSL complex, which is restricted to the nucleus during interphase, after the nuclear envelope breakdown redistribute to spindle structures where they are thought to play mitotic functions.

The *Drosophila* Nonspecific Lethal (NSL) complex is the fly counterpart of the KANSL complex. It includes seven conserved subunits: Nsl1/Wah (KANSL1), Dgt1/Nsl2 (KANSL2), Rcd1/Nsl3 (KANSL3), Rcd5 (MCRS1), MBD-R2 (PHF20), Wds (WDR5) and Mof (MOF/KAT8). MBD-R2, Rcd5, Nsl1, and Mof colocalize in the interbands of polytene chromosomes of third instar larvae, and MBD-R2 physically interacts with the histone modifying complexes Trx/MLL and the Nup98 nucleoporin [10, 11]. Wds is not only a member of the NSL complex, but is also part of several chromatin complexes including the ATAC histone acetyltransferase and the Trx/MLL histone methyltransferase [9, 12, 13]. The NSL complex associates with the promoters of more than 4,000 housekeeping genes, indicating that it acts as a major transcriptional regulator [10, 12, 14].

As in the case of their human counterparts, depletion of the NSL complex members results in mitotic defects. Genome-wide RNAi screens performed in S2 cells showed that Dgt1, Rcd1 and Rcd5 control mitotic centrosome behavior. Dgt1 (diminished γ-tubulin 1) is required for γ-tubulin recruitment, Rcd1 (Reduction in Cnn dots 1) for centriole duplication, and Rcd5 (Reduction in Cnn dots 5) for both centriole duplication and pericentriolar material (PCM) recruitment at the centrosomes [15, 16]. However, the mechanisms through which Dgt1, Rcd1 and Rcd5 regulate centriole duplication and centrosome maturation are currently unknown. Another RNAi-based screen in S2 cells showed that Rcd1 and MBD-R2 are required for mitotic chromosome segregation, but the mechanisms leading to this phenotype are also unknown [17].

Here, we analyzed the mitotic phenotypes caused by Rcd1, Rcd5, MBD-R2 or Wds depletion in greater detail. Cells depleted of these proteins showed a common defect in centrosome duplication, confirming the centrosome phenotype described earlier for *Rcd1* and *Rcd5* RNAi cells [16]. However, we found that *Rcd1*, *Rcd5* and *MBD-R2* RNAi cells, and to a lesser extent *wds* RNAi cells, also exhibit defects in chromosome alignment and segregation. Accordingly, Rcd1-, Rcd5- and MBD-R2-depleted cells displayed reduced levels of both Cid and Ndc80. Cid is a centromere-specific histone variant homologous to CENP-A that is required for kinetochore assembly [18], and Ndc80 is the protein that directly mediates MT attachment to the kinetochore [19]. We generated stable S2 cell lines for the inducible expression of Rcd1-GFP, Rcd5-GFP, MBD-R2-GFP or Wds-GFP. We show that these tagged proteins largely rescue the mitotic phenotype caused by RNAi-mediated depletion of their endogenous counterparts, and localize to the nucleus of interphase cells, as expected for transcription factors. However, during mitosis each protein relocalizes to a specific set of mitotic structures, including the centrosomes, the kinetochores and different regions of the midbody. Our results suggest that the common mitotic phenotypes generated by the depletion of the four NSL components are primarily due to reduced transcription of genes encoding centromere/kinetochore components and genes required for centriole duplication. The different mitotic localizations of Rcd1, Rcd5, MBD-R2 and Wds further suggest that these proteins might have acquired some direct mitotic roles.

## Results

### *Rcd1*, *Rcd5*, *MBD-R2* and *wds* RNAi cells are defective in centrosome behavior

**Fig 1:**
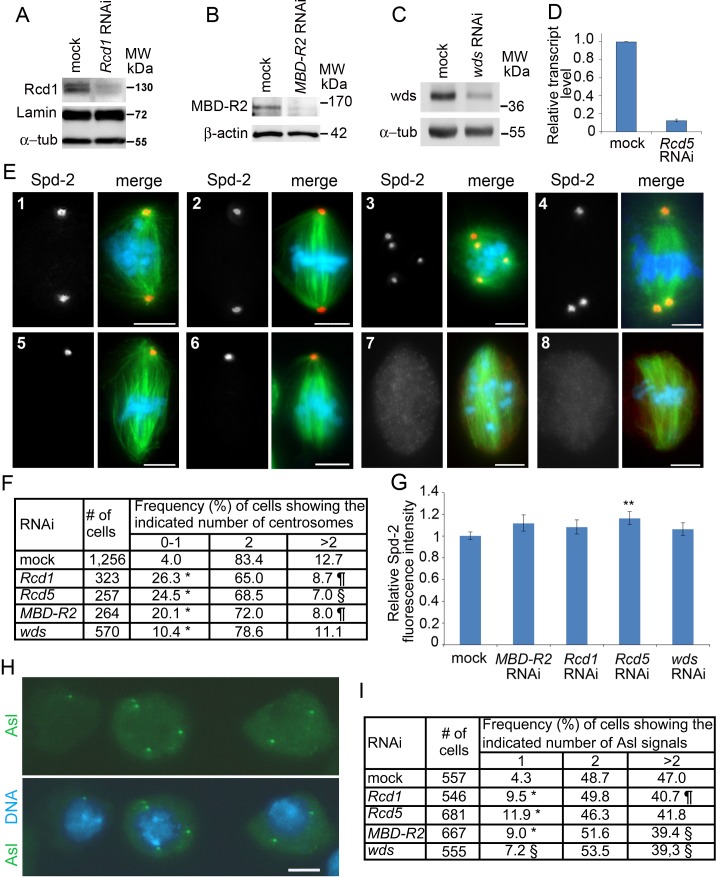
RNAi against *Rcd1*, *Rcd5*, *MBD-R2* and *wds* impairs centriole duplication. (A, B, C) Western blots of S2 cell extracts showing that RNAi against *Rcd1*, *MBD-R2* or *wds* strongly reduces the levels of the corresponding proteins; the two bands detected by the anti-Rcd1 antibody likely correspond to different isoforms of this protein (FlyBase.org). Lamin Dm0 (Lamin), α-tubulin and β-actin are loading controls. (D) RT-qPCR results showing that RNAi against *Rcd5* strongly reduces the level of its transcripts relative to a mock control that is set to 1. (E) Examples of cells showing different centrosome numbers. (E1, E2) Prometaphase (E1) and metaphase (E2) showing two regular centrosomes; (E3, E4) cell with basic karyotypes with 4 (E3) and 3 (E4) centrosomes; (E5-E8) prometaphases and metaphases showing a single centrosome (E5, E6) or no centrosomes (E7, E8). Scale bars, 5 μm. (F) Frequencies of prometaphases and metaphases with the indicated number of centrosomes; ¶, § and *, significantly different from control in the Chi-square test with p < 0.05, p < 0.01 and p < 0.001, respectively. (G) Quantitation of Spd-2 fluorescence in cells with two centrosomes (at least 106 centrosomes examined for each treatment). The mean fluorescence intensities measured in *Rcd1*, *MBD-R2* and *wds* RNAi cells are not significantly different from control in the Wilcoxon Signed-Rank Test; in *Rcd5* RNAi cells the centrosome fluorescence intensity is significantly higher than in control with p < 0.01 (**). (H) Example of interphase nuclei expressing Asl-GFP and showing (from left to right) 1, 4 and 2 Asl signals. Scale bar, 5 μm. (I) Frequencies of interphase cells with the indicated number of Asl-GFP signals; ¶, § and *, significantly different from control in the Chi-square test with p < 0.05, p < 0.01 and p < 0.001, respectively.

We first analyzed the centrosomal phenotype of S2 cells exposed to *Rcd1*, *Rcd5*, *MBD-R2* or *wds* double stranded RNAs (dsRNAs) for 5 days. To check for RNAi efficiency we performed Western blotting of cell extracts using antibodies directed to Rcd1, MBD-R2 or Wds. These assays showed that the antibodies specifically recognize bands of the expected molecular weights (MWs) that are strongly reduced after RNAi (Fig 1A–1C). The efficiency of RNAi against *Rcd5* was demonstrated by RT-qPCR, which revealed a drastic reduction of the *Rcd5* transcript level (Fig 1D). RNAi cells were fixed and stained for DNA, tubulin and the centrosomal marker Spd-2 [20, 21]. We examined only cells that appeared to have the basic karyotype of the S2 cells (~ 12 chromosomes [22]) and did not consider cells that are clearly “polyploid” that are common in S2 cell cultures. However, several S2 cells with the basic karyotype have either a single centrosome or more than two centrosomes (Fig 1E).

To ascertain whether depletion of Rcd1, Rcd5, MBD-R2 or Wds affects centrosome structure and duplication, we scored the Spd-2 signals in prometaphase and metaphase cells from 3 independent experiments. In this analysis, we counted cells showing no centrosomes, a single centrosome, two centrosomes, or more than 2 centrosomes. We did not subdivide the cells with more than 2 centrosomes in subclasses (i.e. cells with different centrosome numbers), because multiple centrosomes tend to overlap or cluster at the spindle poles and are therefore difficult to count. In addition, we did not take into account cells showing very small Spd-2 signals, because it was unclear whether they represented examples of PCM fragmentation or were produced by immunostaining artifacts.

In *Rcd1*, *Rcd5*, *MBD-R2* and *wds* RNAi cells, the frequencies of cells showing 0–1 centrosomes were significantly increased compared to controls, although in Wds-depleted cells this increase was more modest that in the other RNAi cells (Fig 1F). The frequency of cells with more than two centrosomes was either significantly decreased (*Rcd1*, *Rcd5*, *MBD-R2* RNAi cells) or unchanged (*wds* RNAi) compared to control (Fig 1F). We also measured the fluorescence intensity of Spd-2 signals in prometaphase and metaphase cells showing two centrosomes; we found that in *Rcd1*, *MBD-R2* and *wds* RNAi cells the average fluorescence of these signals is not significantly different from controls, while in Rcd5*-*depleted cells it was slightly but significantly higher (p < 0.01; Wilcoxon Signed-Rank Test) than in control (Fig 1G and S1 Data). Consistent with these findings, we found that the asters of these RNAi cells are indistinguishable from those of control cells. We note that the results on *Rcd1* RNAi cells are fully consistent with those of Dobbelaere *et al*. [16], who showed that depletion of Rcd1 reduces the centrosome number without affecting PCM recruitment. Dobbelaere *et al*. also showed that Rcd5 depletion negatively affects centrosome duplication but also reduces Cnn recruitment at centrosomes. We confirmed that Rcd5 deficiency reduces centrosome number but we found that it leads to a small increase in the fluorescence intensity of centrosome-associated Spd-2. We do not know the reasons for this apparent discrepancy but it is possible that the centrosomes of Rcd5-depleted cells are defective in Cnn recruitment but have normal or slightly increased ability to associate with Spd-2.

In *Drosophila* S2 cells, which often contain multiple centrosomes and can divide even in the absence of centrosomes, centrosome loss could arise either through defective centrosome separation during prophase or defective centriole duplication during interphase [16, 23]. Failure in centrosome separation in prophase would lead to formation of a bipolar spindle with two centrosomes (each containing a pair of centrioles) at one pole and no centrosome at the other. Because S2 cells can assemble fully functional monastral spindles [24], a defect in prophase centrosome separation is expected to ultimately lead to an increase in both cells with zero and with more than two centrosomes. In the case of defective centriole duplication during interphase, the expectation is instead an increase in cells with 0–1 centrosomes and a decrease in cells with more than 2 centrosomes. Thus, our results (Fig 1F) are consistent with the second alternative, and suggest that depletion of the NSL components primarily impairs centriole duplication.

To confirm and extend these results, we counted the number of centrioles in interphase cells depleted of Rcd1, Rcd5, MBD-R2 or Wds. As centriole marker we decided to use Asterless (Asl), a protein that is thought to link the centriole wall with the PCM [25–27]. However, preliminary tests with antibodies directed to Asl or to other centriolar components revealed that in our hands they do not stain the centrioles in all interphase cells. We thus constructed an S2 cell line that expresses Asl-GFP under the control of the copper-inducible *Metallothionein A* (*MtnA*) promoter, and used it in the RNAi experiments against the NSL genes. As Asl overexpression causes centriole overduplication during the S phase [26], we limited the impact of this event by treating both RNAi and control cells with CuSO_4_ for only 12 hours before fixation (the cell cycle of S2 cells lasts approximately 24 hours). We stained control and RNAi cells with an anti-GFP antibody and scored them for the number of Asl signals present in interphase cells (Fig 1H). We did not take into account cells with zero signals because they could be cells in which the Asl-GFP expression was not sufficiently strong to mark the centrioles. RNAi-mediated depletion of Rcd1, Rcd5, MBD-R2 or Wds resulted in significant increases in interphase cells showing a single Asl signal compared to control. We also found significant decreases in cells with more than 2 signals in *Rcd1*, *MBD-R2* and *wds* RNAi cells; the frequency of *Rcd5* RNAi cells with multiple centrioles was also reduced compared to control but not significantly (p = 0.07) (Fig 1H and 1I). Collectively, these results reinforce the conclusion that the NSL components are required for centriole duplication.

### *Rcd1*, *Rcd5* and *MBD-R2* RNAi cells are severely defective in chromosome segregation

**Fig 2:**
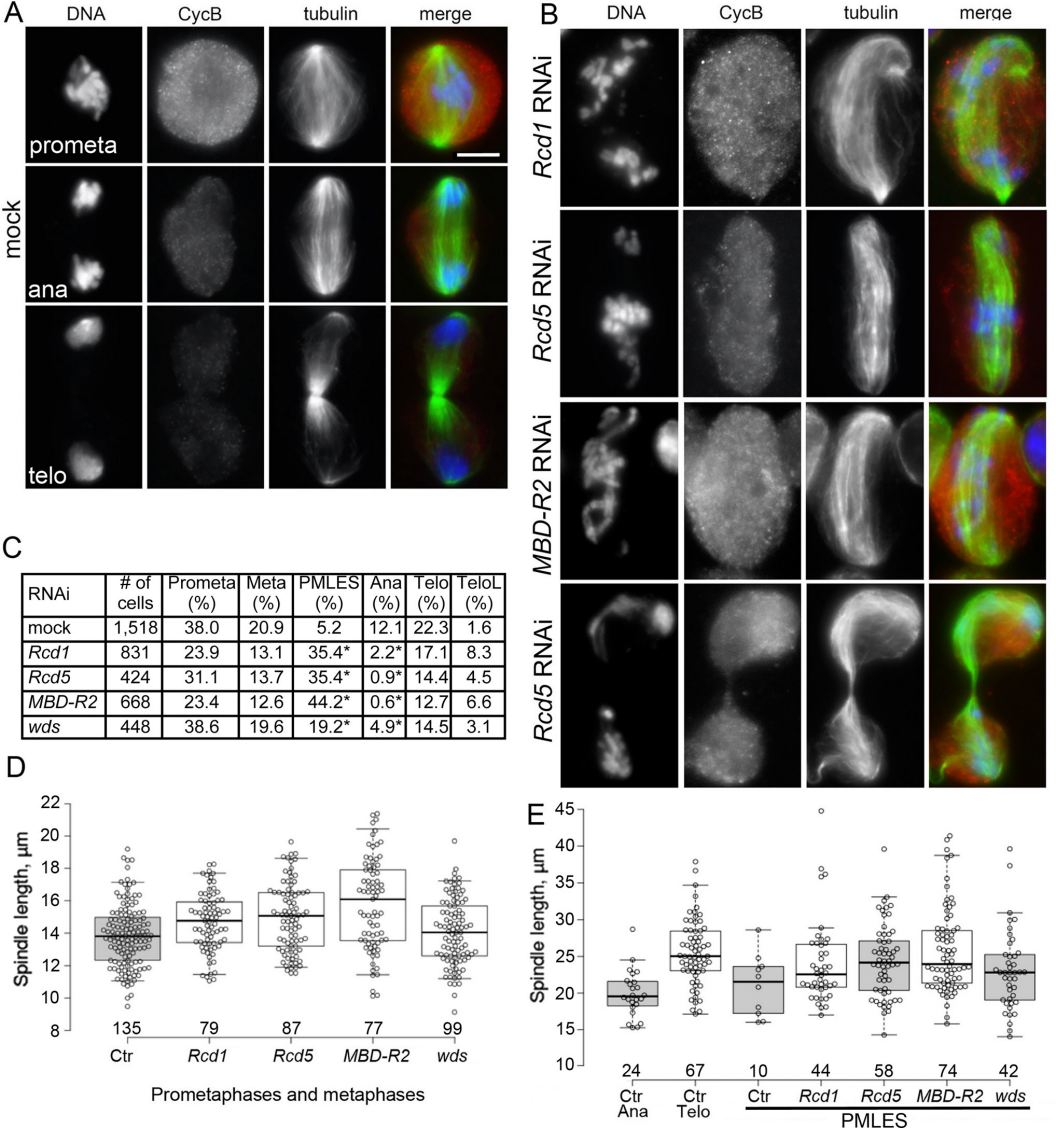
RNAi-mediated depletion of Rcd1, Rcd5, MBD-R2 or Wds disrupts chromosome segregation. (A-B) Mitotic figures observed in mock-treated control cells (A), and in *Rcd1*, *Rcd5* or *MBD-R2* RNAi cells (B) stained for DNA (blue), α-tubulin (green) and Cyclin B (CycB; red). The cells shown in (B) are PMLES, namely prometaphase-like cells with elongated anaphase-like or telophase-like spindles showing high Cyclin B levels. Note that the cells with the arched anaphase-like spindles contain chromosomes comprised of both sister chromatids; see text for detailed explanation and S1 Fig for additional PMLES. Scale bar for panels A and B, 5 μm. (C) Frequencies of mitotic figures observed in *Rcd1*, *Rcd5*, *MBD-R2* and *wds* RNAi cells and in mock-treated control cells. Prometa, prometaphases; Meta, metaphases; PMLES, as those shown in panel (B) and S1 Fig; Ana, anaphases; Telo, telophases; TeloL, telophases with lagging chromosomes. The Telo and TeloL classes could include real telophases or PMLES with telophase-like spindles and decondensed chromosomes (see panel B of this figure and S1D Fig). Scale bar, 5 μm. (D) Box-Whisker plots showing the length of prometaphase/metaphase spindles of mock-treated control (Ctr) and RNAi cells (*Rcd1*, *Rcd5*, *MBD-R2* and *wds*). In *Rcd1*, *Rcd5* and *MBD-R2* RNAi cells, the spindle is significantly longer than in control cells in the Wilcoxon Signed-Rank Test (p < 0.01 in all cases); the spindles of *wds* RNAi cells are not significantly different from control spindles. (E) Box-Whisker plots showing the spindle length of anaphases (Ana), telophases (Telo) and PMLES observed in control (Ctr) cells, and of PMLES seen in *Rcd1*, *Rcd5*, *MBD-R2* and *wds* RNAi cells. The PMLES spindle length in *Rcd5* and *MBD-R2* RNAi cells is not significantly different from the length of control telophase spindles in the Wilcoxon Signed-Rank Test, while the PMLES spindles of *Rcd1* and *wds* RNAi cells are slightly but significantly shorter than the telophase spindles of control cells (p < 0.01) (see also S2 Data). The numbers below each box shown in D and E indicate the numbers of spindles measured.

An analysis of mitotic division in several independent experiments revealed that Rcd1-, Rcd5- and MBD-R2-depleted cells exhibit very similar phenotypes. They display an approximately ten-fold reduction in the frequency of anaphases compared to controls and very high frequencies of PMLES (prometaphase-like cells with elongated spindles) figures (Fig 2A–2C). PMLES (formerly called pseudo-ana-telophases, abbreviated with PATs [17]) are peculiar mitotic figures that contain late anaphase/early telophase-like spindles associated with chromosomes that are improperly comprised of both sister chromatids and are usually scattered along the spindles; in some cases PMLES exhibit central spindle-like structures and irregular cytokinetic rings (Fig 2B and S1 Fig; see also refs. [17, 28]). Notably, several PMLES exhibit arched spindles, which are presumably the consequence of an excessive spindle elongation within the constraints imposed by the plasma membrane. Despite their ana-telophase-like spindle structure, PMLES contain high levels of Cyclin B, which is normally degraded at the beginning of anaphase (Fig 2A and 2B). Thus, PMLES appear to be in a pre-anaphase stage as far as sister chromatid separation and Cyclin B degradation are concerned, but they are nevertheless permissive of typical telophase events, such as central spindle assembly and initiation of cytokinesis.

To further characterize the PMLES, we measured the length of their spindle axis and compared it with the length of the other mitotic figures. In *Rcd1*, *Rcd5* and *MBD-R2* RNAi cells the prometaphase and metaphase spindles were morphologically normal but slightly longer (~10%) than their control counterparts (Fig 2D, S2 Data). PMLES spindles of RNAi cells were instead substantially longer than prometaphase/metaphase spindles (~ 60%) and anaphase spindles (~ 25%) (Fig 2D and 2E). The average length of the PMLES spindles was either slightly shorter or similar to the length of the spindles of control telophases (Fig 2E), indicating that most PMLES can attain the maximum spindle elongation that is normally achieved by S2 cells. The slight increase in the prometaphase and metaphase spindle length observed in RNAi cells is likely to reflect the presence of some cells in the initial stages of evolution towards a PMLES configuration.

Although *Rcd1*, *Rcd5* and *MBD-R2* RNAi cells exhibit very low anaphase frequencies (Fig 2C), they show relatively high frequencies of mitotic figures with telophase-like spindles, characterized by the presence of a constricted central spindle and decondensed chromosomes at the cells poles; about a third of these cells displayed lagging chromosomes between the cell poles (Fig 2B and S1D Fig). Because the chromosomes in these cells are decondensed, it is not possible to discern whether they contain one or two sister chromatids. Nonetheless, we favor the idea that many of the “telophases” observed in Rcd1-, Rcd5- and MBD-R2-depleted cells are in fact PMLES that managed to progress further through the mitotic process and undergo chromosome decondensation as normally occurs in telophase. Regardless of the nature of these telophase-like cells, it is clear that Rcd1, Rcd5 and MBD-R2 are all required for sister chromatid separation and chromosome segregation.

Cells depleted of Wds displayed a mitotic phenotype qualitatively similar but quantitatively milder than that observed in Rcd1-, Rcd5- or MBD-R2-deficient cells (Figs 1F and 2C-2E). Thus, although the degree of RNAi-mediated depletion of the Wds protein is comparable to that observed for the other NSL proteins (Fig 1C), in *wds* RNAi cells both the centrosome and chromosome segregation phenotypes are substantially milder than those observed in *Rcd1*, *Rcd5* or *MBD-R2* RNAi cells.

### Rcd1, Rcd5 and MBD-R2 are required for centromere and kinetochore formation

Collectively our findings indicate that Rcd1-, Rcd5- and MBD-R2-depleted cells are severely defective in chromosome segregation. Depletion of Wds also perturbed chromosome segregation but caused a relatively mild defect. This chromosome segregation phenotype cannot be ascribed to centrosome defects, as abundant evidence indicates that *Drosophila* mitotic spindle assembly and functioning does not require the centrosomes [15, 17, 29–31].

We have previously observed frequent PMLES in S2 cells depleted of the centromere-specific histone H3 Cid/Cenp-A or the kinetochore components Ndc80, Nuf2 and Kmn1 [17, 32]. PMLES have been also observed in S2 cells depleted of Mast/Orbit that has a role in MT-kinetochore attachment [33], or depleted of the Klp67A kinesin-like protein, which represses MT plus end growth and is required for proper MT binding to kinetochores [32]. More recently, PMLES were observed in S2 cells depleted of the Sf3A2 and Prp31 splicing factors that have direct roles in MT-kinetochore interactions [28]. These results suggest that the PMLES found in *Rcd1*, *Rcd5* and *MBD-R2* RNAi cells are a consequence of a defective kinetochore-MT attachment.

**Fig 3:**
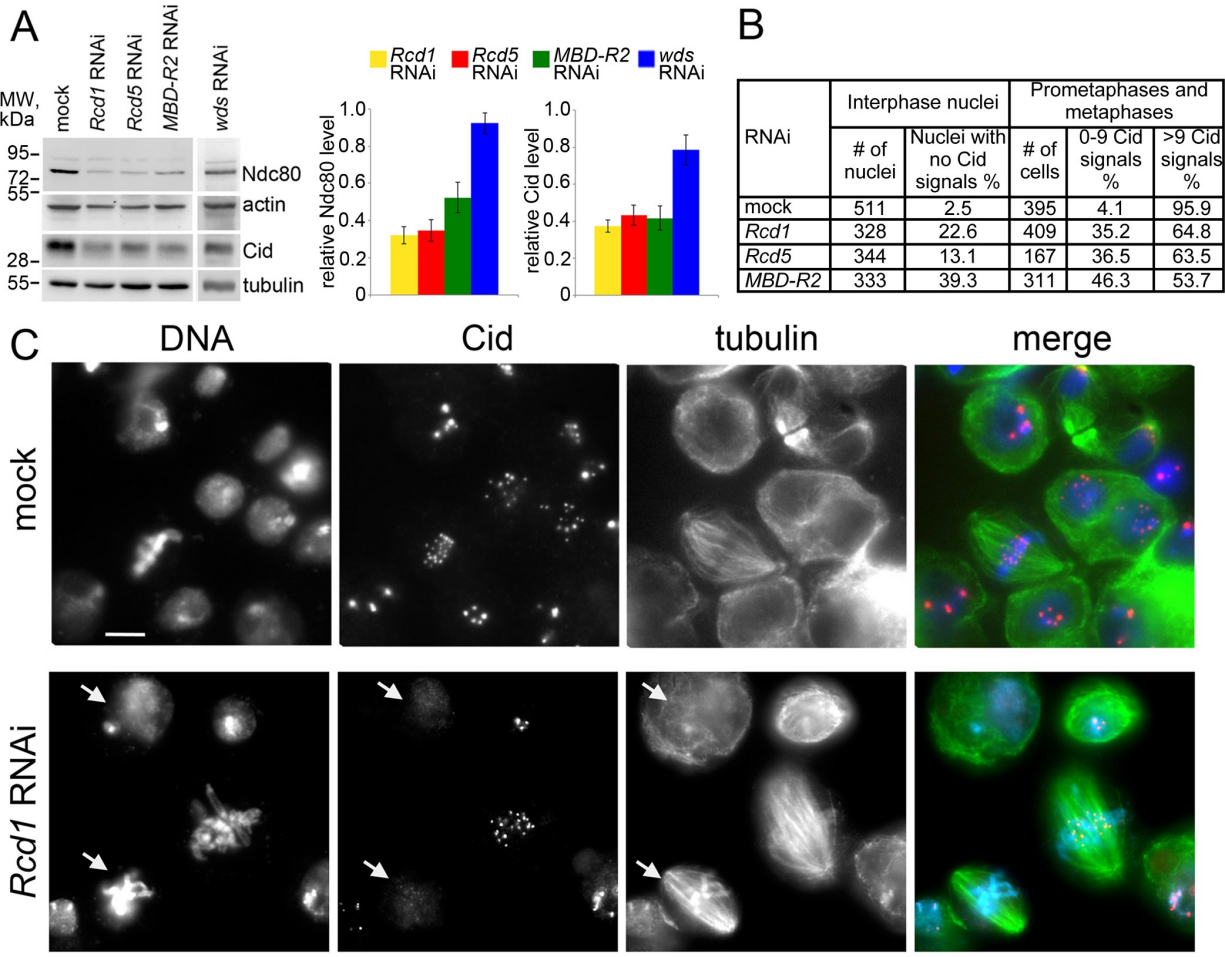
RNAi-mediated depletion of Rcd1, Rcd5 or MBD-R2 reduces the levels of Cid and Ndc80. (A) Western blotting analysis and band intensity quantification relative to the control from 3 independent experiments showing that in *Rcd1*, *Rcd5*, *MBD-R2* and *wds* RNAi cells the Cid level is reduced to 38%, 43%, 42% and 78% of the control level, respectively. In cells depleted of Rcd1, Rcd5 or MBD-R2, Ndc80 is reduced to 32%, 35% and 53% of the control level, respectively; in cells depleted of Wds the Ndc80 level is not changed compared to control. β-actin and α-tubulin are loading controls (see also S3 Data). (B) Quantification of the Cid signals in interphase and mitotic cells from control and Rcd1-, Rcd5- or MBD-R2-depleted cells. The frequencies of interphase nuclei with no Cid signals, and prometaphases/metaphases with 0–9 signals observed in *Rcd1*, *Rcd5* or *MBD-R2* RNAi cells are significantly higher than those detected in mock-treated cells (Chi-square test; in all cases p < 0.001). (C) Examples of interphase and dividing cells from mock-treated control and *Rcd1* RNAi cultures stained for DNA (blue), Cid (red) and α-tubulin (green). Note that nearly all control cells display clear Cid signals; in contrast, some of the Rcd1-depleted interphase and dividing cells exhibit extremely weak or no Cid signals (arrows). Scale bar, 5 μm.

To test this possibility, we first analyzed the effects of Rcd1, Rcd5, MBD-R2 or Wds depletion on the intracellular concentration of the core centromere component Cid/CenpA that is required for kinetochore assembly [18], and Ndc80 that is directly responsible for MT-kinetochore attachment [19, 34–36]. Western blotting analysis showed that in Rcd1-, Rcd5- and MBD-R2-depleted cells Cid is considerably reduced compared to controls (32%, 36% and 40% of the control level, respectively); the level of Cid was also reduced in Wds-depleted cells but to a lesser extent (78%) (Fig 3A, S3 Data). In addition, Western blotting on extracts from *Rcd1*, *Rcd5* and *MBD-R2* RNAi cells showed substantial reductions of Ndc80 compared to mock-treated cells (32%, 35% and 52% of the control level, respectively), while in *wds* RNAi cells the Ndc80 level was comparable to that of controls (Fig 3A, S3 Data).

To extend the analysis at the subcellular level we focused on *Rcd1*, *Rcd5* and *MBD-R2* RNAi cells, which exhibit strong reductions in the Cid content compared to both control and Wds-depleted cells. The Cid protein is detectable both at the centromeres of mitotic chromosomes and in interphase nuclei; nuclei of control cells exhibit 3–6 foci corresponding to clustered centromeres (Fig 3B). In *Rcd1*, *Rcd5* and *MBD-R2* RNAi cells, the frequencies of interphase nuclei devoid of Cid signals were significantly higher than in controls (Fig 3B and 3C). We also examined prometaphases and metaphases with a basic karyotype for the presence of Cid signals. In general, RNAi cells displayed Cid signals of lower intensity compared to controls; they also showed a great variability in the number of detectable signals, ranging from zero to more than 20 signals (in a cell with 12 chromosomes the maximum number of Cid signals is 24). In Rcd1-, Rcd5- and MBD-R2-depleted cells, the frequencies of cells with 0–9 signals were drastically increased compared to controls, in which this type of cells are virtually absent (Fig 3C).

### Rcd1, Rcd5 and MBD-R2 are only partially interdependent

**Fig 4:**
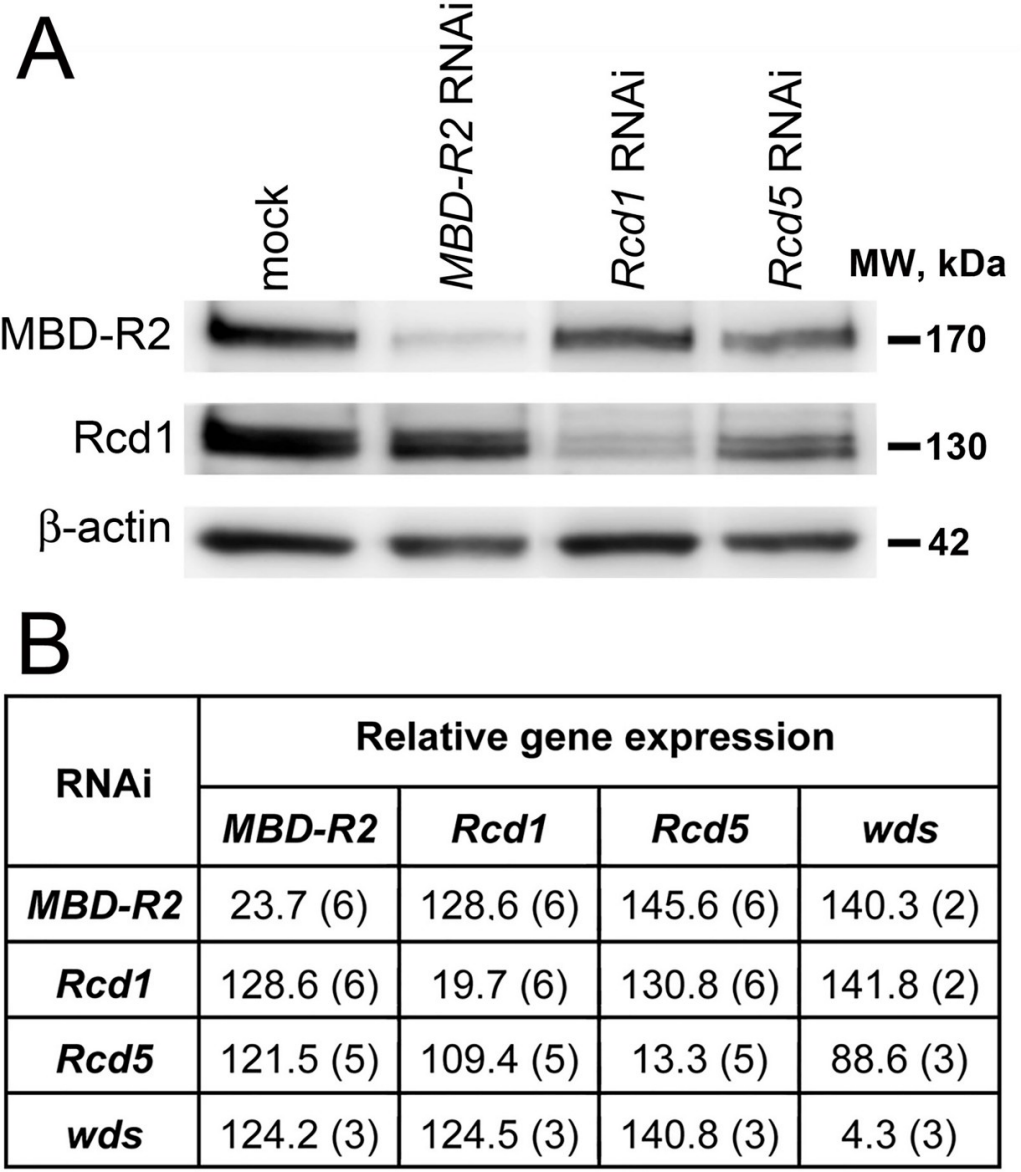
Partial interdependence between Rcd5 and Rcd1. (A) Western blot showing that RNAi-mediated depletion of *Rcd5* leads to a small reduction in Rcd1 and an even smaller reduction in MBD-R2 levels. MBD-R2 depletion does not substantially affect the Rcd1 level. β-actin is a loading control. (B) Average gene expression relative to control measured by RT-qPCR after RNAi against *Rcd1*, *Rcd5*, *MBD-R2*, or *wds*. The numbers between brackets indicate numbers of biological replicates.

The similarity of the mitotic phenotypes observed in Rcd1-, Rcd5- and MBD-R2-depleted cells might be a consequence of an interdependence of these proteins. Indeed, it has been previously shown that depletion of Rcd5 in salivary glands leads to a severe reduction in Rcd1, while Rcd1 depletion results only in a slight reduction in Rcd5 [10]. Similar results were obtained in SL-2 cells, where RNAi-mediated depletion of Rcd5 caused a reduction in Rcd1, whereas Rcd1 depletion did not substantially affects the Rcd5 level [10]. In both salivary glands and SL-2 cells, MBD-R2 depletion did not affect the stability of the other members of the complex [10], while the Wds levels were similar to those of controls in *Rcd1*, *Rcd5* or *MBD-R2* RNAi cells [10]. We found that RNAi against *Rcd5* leads to a small reduction in Rcd1 also in the S2 cell line used here. *Rcd5* RNAi cells displayed a small reduction in MBD-R2, while MBD-R2 depletion did not substantially affect the Rcd1 level (Fig 4A). Consistent with these results, RT-qPCR showed that RNAi against each of the four NSL genes studied here does not substantially affect transcription of the others, which, with the exception of *wds* upon *Rcd5* RNAi, are expressed at slightly higher rates compared to control (Fig 4B). These results suggest that the phenotypes observed in Rcd1-, Rcd5-, MBD-R2- and Wds-depleted cells are largely due to the deficiency of each individual factor and do not reflect interdependencies among these proteins.

### Rcd1, Rcd5, MBD-R2 and Wds localize to specific regions of the mitotic apparatus

In the attempt of detecting a mitotic localization of the NSL subunits, we first tested the commercial anti-MBD-R2 and anti-Wds antibodies and our anti-Rcd1 mouse antibody. All these antibodies specifically recognize bands of the expected MWs that are strongly reduced in RNAi cells (Figs 1A–1C and 4A). However, immunostaining using the same antibodies showed that the anti-MBD-R2 and anti-Wds antibodies stain weakly only the interphase nuclei but do not decorate any mitotic structure, while the anti-Rcd1 antibody did not work at all in indirect immunofluorescence.

**Fig 5:**
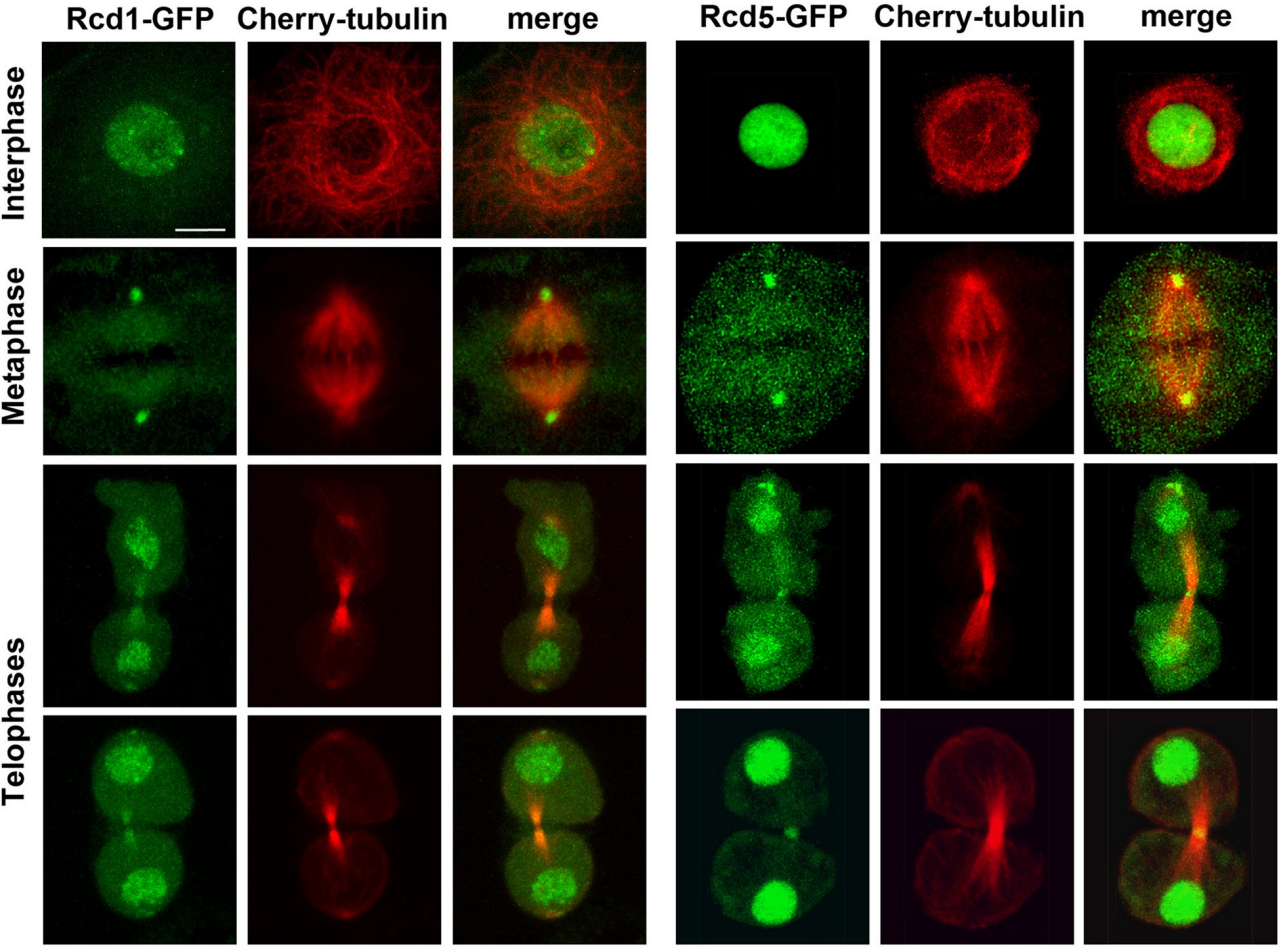
Localization of Rcd1-GFP and Rcd5-GFP in live interphase and dividing S2 cells. The cells express Cherry-tubulin and either Rcd1-GFP or Rcd5-GFP. Note that in metaphase cells Rcd1-GFP localizes to the center of the MT asters in a position that is normally occupied by the centrosomes. Rcd1-GFP also localizes at both sides of the dark zone of the midbody of telophase cells. Rcd5-GFP localizes to the center of the asters in metaphases and in the dark zone of the midbody of telophase cells. Scale bars, 5 μm for all panels.

**Fig 6:**
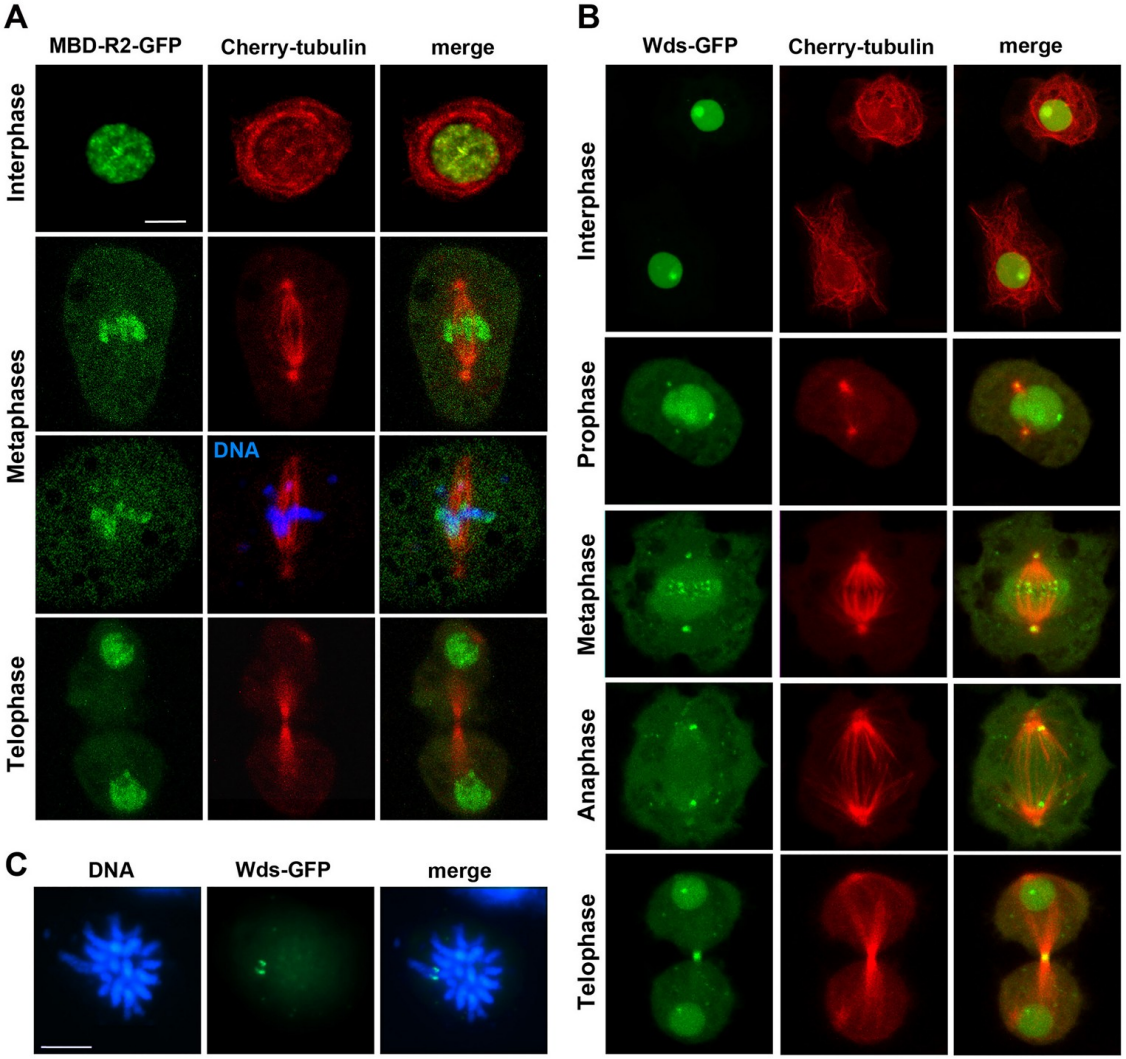
Localization of MBD-R2-GFP and Wds-GFP in live interphase and dividing S2 cells. The cells express Cherry-tubulin and either MBD-R2-GFP or Wds-GFP. (A) MBD-R2-GFP is associated with the chromosomes and fails to accumulate in either the centrosomes or the midbody of telophase cells. The cell in the third row is also stained for DNA (blue) with the vital die Hoechst 33342. (B) In interphase cells, Wds-GFP localizes to the nucleus and is enriched in a discrete nuclear region. In prophases and metaphases, Wds-GFP is enriched at the center of the asters but this localization is rarely observed in anaphases and telophases. In metaphases, Wds-GFP is transiently associated with structures that are likely to be the kinetochores. In telophases, Wds-GFP is consistently enriched at the midbody dark zone. Finally, in all mitotic phases Wds-GFP is tightly associated with a discrete chromosomal region. (C) Fixed prometaphase cell immunostained with an anti-GFP antibody and counterstained with DAPI. Note that the GFP signal is located to a discrete region of an acrocentric chromosome that is likely to be the X chromosome. See text for further explanation. Scale bar, 5 μm.

We then generated stable cell lines expressing Cherry-tubulin and any of Rcd1-GFP, Rcd5-GFP, MBD-R2-GFP or Wds-GFP tagged proteins, all under the control of the copper-inducible *MtnA* promoter. We exposed these cells for 12–14 hours to different concentrations of CuSO_4_ (0.1, 0.25, 0.4 and 0.5 mM) and examined them under a confocal fluorescence microscope. All GFP-tagged proteins showed strong nuclear signals but also displayed clear but different mitotic localizations (Figs 5 and 6). In prometaphase and metaphase cells, Rcd1-GFP and Rcd5-GFP proteins were no longer associated with chromatin, but accumulated at the centers of the asters/centrosomes and were occasionally weakly enriched at the spindle area. In anaphase and telophase cells, the accumulation at the centrosomes was reduced compared to the previous mitotic phases. In telophase cells, Rcd1-GFP and Rcd5-GFP concentrated in the daughter nuclei as expected for transcription factors, but were also enriched at the midbody, the structure that connects the two daughter cells during late telophase and cytokinesis (Fig 5). The midbody contains bundled antiparalled MTs with their plus ends overlapping at the center of the structure. The MT overlapping area is often dark (dark zone) after staining with anti-tubulin antibodies because it is enriched in proteins that block antibody binding to tubulin [37]. We see a dark zone also in living cells expressing Cherry-tubulin, most likely because the same proteins that prevent antibody binding quench the Cherry-tubulin fluorescence. Interestingly, while Rcd1 was excluded from the dark zone and enriched at both sides of this region, Rcd5 was specifically accumulated in the dark zone at the center of the midbody (Fig 5).

After CuSO_4_ induction, MBD-R2-GFP and Wds-GFP were strongly enriched in interphase nuclei but showed very different localization patterns in mitotic cells. MBD-R2-GFP localized exclusively at the chromosomes and did not show accumulations at either the centrosomes or the midbody (Fig 6A). In contrast, Wds-GFP was enriched at several mitotic structures (Fig 6B and 6C). In all mitotic phases, Wds-GFP accumulated in a discrete region of a specific chromosome. This GFP signal was also detected in both telophase and interphase nuclei of living cells, and was sufficiently strong to be detected in fixed cells with well spread chromosomes (Fig 6B and 6C). This allowed us to localize the Wds-GFP accumulation on a specific region of an acrocentric chromosome characterized by a highly DAPI-fluorescent pericentric heterochromatin (Fig 6C). The DAPI staining pattern of this chromosome and the localization of the GFP signal along the chromosome suggest that Wds might associate with the nucleolus organizer of the X chromosome in both mitotic cells and interphase nuclei [22]. We also observed an enrichment of Wds-GFP at the centrosomes; this enrichment was clearly visible in most prophase, prometaphase and metaphase cells, but was hardly detectable in anaphases and telophases. In addition, in most late prometaphase and metaphase cells, Wds-GFP was enriched at structures that are likely to correspond to the centromeres/kinetochores (Fig 6B). This localization is transient, and was never observed in the other mitotic phases. In all telophase cells, Wds-GFP was accumulated at the midbody dark zone.

The cells shown in Figs 5 and 6 were treated for 12–14 hours with 0.4 mM CuSO_4_, as this is the optimal concentration for a clear visualization of both the GFP-tagged protein and Cherry-tubulin. However, we were able to see mitotic accumulations of GFP-tagged proteins also after 12–14 hours induction with 0.1 mM CuSO_4_. Importantly, at all CuSO_4_ concentrations, we consistently observed the same localization patterns as those shown in Figs 5 and 6. Thus, the mitotic localization of each protein appears to be a characteristic feature of the protein independent of its intracellular concentration.

To obtain further insight into this issue, we also performed a Western blotting analysis to determine the levels of the endogenous and the GFP-tagged forms of Rcd1, MBD-R2 and Wds after CuSO_4_ induction (S2 Fig). We could not carry out this analysis for cells expressing Rcd5-GFP due to the unavailability of an anti-Rcd5 antibody. These experiments showed that MBD-R2-GFP and its endogenous counterpart were expressed at similar levels after induction with 0.1 or 0.25 mM CuSO_4_, while induction with 0.5 mM CuSO_4_ led to a limited MBD-R2-GFP overexpression (S2A Fig, S4 Data). In *MtnA-Rcd1-GFP*-bearing cells, the endogenous and the GFP-tagged protein were expressed at similar levels after induction with 0.1 mM CuSO_4,_ but the GFP protein was 2.5- and 3.9-fold more abundant than the normal protein after induction with 0.25 and 0.5 mM CuSO_4,_ respectively (S2B Fig, S4 Data). Wds-GFP was expressed at relatively high levels at all CuSO_4_ concentrations, with the tagged protein showing expression levels 3-4-fold higher than that of the corresponding endogenous protein (S2C Fig, S4 Data). These results strongly suggest that Rcd1 and MBD-R2 localizations are independent of the concentration of the individual proteins.

The lack of biochemical data on Rcd5-GFP and the fact the Wds-GFP is 3 to 4 times more abundant than the endogenous protein do not permit us to exclude that the mitotic localizations of these proteins could be partially affected by their intracellular quantity. However, an analysis of live cells expressing either Rcd5-GFP or Wds-GFP strongly suggests that this is not the case. In cells induced with 0.1 mM CuSO_4_, there is great variability in the levels of the GFP proteins expressed by the individual dividing cells. Nonetheless, regardless their degree of GFP fluorescence, all mitotic cells showed the same Rcd5-GFP- or Wds-GFP-specific accumulations (see Figs 5 and 6), suggesting that the mitotic localization of these proteins is largely independent of their intracellular concentration.

We also fixed the cells treated for 12 hours with either 0.1 or 0.5 mM CuSO_4_ and stained them with anti-GFP and anti-tubulin antibodies. Cells expressing Rcd1-GFP, Rcd5-GFP, MBD-R2-GFP or Wds-GFP fixed with standard formaldehyde- and paraformaldehyde-based procedures (see Materials and Methods) showed an evident GFP staining of interphase nuclei. However, the localization of these proteins on the mitotic apparatus was fixation-dependent but independent of the concentration of CuSO_4_. In Rcd1-GFP and Rcd5-GFP expressing cells, we did not detect any clear accumulation of the tagged proteins at either the centrosomes or the central spindles (S3 Fig). This prevented double immunostaining experiments aimed determining whether Rcd1 and Rcd5 precisely colocalize with the centrosomes. Previous work has shown that both Rcd1 and Rcd5 are required for centriole duplication in S2 cells but failed to detect accumulations of these proteins at the centrosomes [16]. However, in this study cells expressing GFP-tagged Rcd1 or Rcd5 were fixed with 4% paraformaldehyde, a treatment that likely disrupted centrosomal localization of the GFP-tagged proteins [16]. In contrast, fixed cells expressing MBD-R2-GFP showed a strong and specific enrichment of the tagged protein at the chromosomes (S3 Fig). Lastly, after fixation, Wds-GFP was consistently enriched at a specific chromosomal region in metaphase and anaphase cells and at the dark zone of the midbody during telophase. The fixation-resistant localization of Wds on a discrete chromosomal region is a likely example of mitotic chromosome bookmarking. Such bookmarking occurs when transcription factors remain associated with chromosomes during mitosis so as to facilitate reactivation of a subset of genes in the subsequent cell cycle [38].

The observation that the GFP-tagged components of the NSL complex exhibit different localization patterns during mitosis raises the question of whether these proteins have the same functions as their non-tagged counterparts. To address this question we performed RNAi using dsRNAs that target only the 5ʹ and 3ʹ untranslated regions (UTRs) of the *Rcd1*, *Rcd5*, *MBD-R2* and *wds* endogenous genes (see S1 Table) but not the coding sequences (CS) of the GFP-tagged transgenes. We specifically asked whether the expression of the GFP-tagged NSL proteins rescues the mitotic effects caused by treatments with the corresponding UTR dsRNAs. In cells expressing the GFP-tagged proteins, dsRNAs targeting of CS resulted in very strong phenotypic effects comparable to those observed in normal cells treated with the same CS dsRNAs (compare Fig 2C with S2 Table). dsRNAs targeting the UTR sequences were less effective than CS dsRNAs in inducing mitotic defects (Fig 2C, S2 Table), consistent with the fact that the CS used for RNAi are considerably longer than the corresponding UTRs (S1 Table). However, when RNAi with UTR dsRNAs was performed in cells expressing the corresponding GFP-tagged proteins (induced by 0.1 mM CuSO_4_; see Materials and Methods) the mitotic effects were substantially milder than those observed in cells that express only the endogenous proteins (S2 Table). In summary, the data reported in the S2 Table show that the GFP-tagged forms of Rcd1, Rcd5, MBD-R2 and Wds rescue the phenotypic defects caused by depletion of the endogenous proteins. This suggests that the GFP-tagged NSL components are largely functional and supports the view that their different localizations during mitosis reflect the normal localizations of their untagged counterparts.

### Rcd1, Rcd5 and MBD-R2 regulate transcription of genes required for kinetochore assembly and centriole duplication

The finding that Rcd1, Rcd5, MBD-R2 and Wds exhibit different localization patterns during mitosis, and yet cause very similar phenotypes when depleted, suggests the hypothesis that these proteins might act together in regulating the expression of mitotic genes. Previous work has shown that at least four components of the NSL complex (Nsl1, Rcd1, Rcd5 and MBD-R2) bind the active promoters of more than 4,000 constitutively expressed genes, suggesting that NSL acts as a complex to specifically upregulate this type of genes [10, 12, 14]. However, it appears that NSL depletion results in diminished expression of only a subset of the genes to which it is bound [12].

To address the possibility that depletion of the NSL components affects mitotic gene transcription, we exploited the published ChIP and gene expression datasets generated in S2 cells [12, 14] to ask whether the NSL complex binds the promoters and regulates the expression of genes encoding (i) centromere and kinetochore proteins (*cid*, *Cenp-C*, *Mis12*, *Nnf1a*, *Nnf1b*, *Kmn1/Nsl1*, *Ndc80*, *Nuf2*, *Spc25*/*Mitch* and *Spc105R/KNL1*), (ii) factors that mediate centriole duplication (*ana2*, *asl*, *SAK*, *Sas-4*, *Sas-6*), and (iii) components of the spindle assembly checkpoint (SAC) machinery (*Mad1*, *mad2*, *Bub1*, *Bub3*, *BubR1*, *Zw10*, *rod*, *Zwilch*, *cmet*, *nudE*). We examined the SAC genes not only as a term of comparison with centromere/kinetochore and centriole duplication genes but also to gather information on whether the SAC is compromised in the absence of functional NSL complex. We found that the promoters of all these genes are bound by at least two components of the NSL complex (Rcd1 and MBD-R2; Table 1 and S3 Table). In addition, interrogation of published datasets [12] revealed that RNAi-mediated Nsl1 depletion results in reduced transcription of most of these genes (Table 1). Among the centromere/kinetochore genes, the strongest reductions in transcription were observed for *Nnf1b*, *Mis12* and *cid*, which were transcribed at 6.1%, 6.9% and 17.5% of the control level, respectively. The genes specifying SAC functions were generally under-transcribed, with *rod* and *Zwilch* showing particularly reduced transcription levels (below 30% the control level), suggesting the SAC could be partially compromised in cells depleted of the NSL components. Finally, all genes required for centriole duplication showed reduced transcription, with *Sas-6*, *Sas-4* and *asl* transcripts reduced to 9.5%, 26.3%, and 30.3% of those of controls, respectively (Table 1).

**Table 1:** (1) Cen, centromere; Kin, kinetochore; SAC, spindle assembly checkpoint; Ced, centriole duplication. (2) ChIP data on Rcd1 and MBD-R2 are from [14]; ChIP data on Nsl1 are from [12]. We did not include *Kmn2* in the table because it was not included in the analyses of [14] and [12]. Transcription factor binding to the gene promoter region is indicated by +; for a quantitative analysis of promoter binding see S3 Table; NA, data not available. (3) Gene expression data are from [12]. (4) The values reported are the means of a number (indicated between brackets) of independent experiments.

Gene	Protein function (1)	Promoter binding measured by ChIP analysis (2)			Expression after *nsl1* RNAi (% of control) (3)	Expression after RNAi against the indicated NSL component (% of control measured by RT-qPCR) (4)			
		Rcd1	MBD-R2	Nsl1		*Rcd1*	*Rcd5*	*MBD-R2*	*wds*
*cid*	Cen	+	+	NA	17.5	50.9 (6)	50.2 (5)	45.7 (5)	73.4 (3)
*Cenp-C*	Cen	+	+	NA	55.6				
*Mis12*	Kin	+	+	NA	6.9	15.4 (2)	26.4 (3)	24.8 (2)	74.5 (3)
*Nnf1a*	Kin	+	+	NA	51.0				
*Nnf1b*	Kin	+	+	+	6.1	16.8 (2)	25.8 (3)	24.2 (2)	78.6 (3)
*Kmn1 (Nsl1)*	Kin	+	+	+	45.7				
*Ndc80*	Kin	+	+	+	37.6	81.2 (4)	65.7 (4)	56.1 (3)	86.5 (3)
*Nuf2*	Kin	+	+	NA	25.4				
*Spc25 (Mitch)*	Kin	+	+	NA	43.6	67.2 (6)	75.9 (5)	74.4 (5)	77.2 (3)
*Spc105R (KNL1)*	Kin	+	+	NA	71.1				
*Mad1*	SAC	+	+	+	41.8				
*mad2*	SAC	+	+	NA	36.3				
*Bub1*	SAC	+	+	NA	107.1				
*Bub3*	SAC	+	+	NA	43.4				
*BubR1*	SAC	+	+	+	37.7				
*Zw10*	SAC	+	+	+	83.9				
*rod*	SAC	+	+	NA	22.4				
*Zwilch*	SAC	+	+	NA	27.2				
*cmet*	SAC	+	+	NA	66.1				
*nudE*	SAC	+	+	+	89.6				
*ana2*	Ced	+	+	+	32.6				
*asl*	Ced	+	+	NA	30.3	39.6 (2)	55.8 (3)	54.3 (2)	103.6 (3)
*SAK*	Ced	+	+	NA	55.1				
*Sas-4*	Ced	+	+	+	26.3	65.8 (2)	65.3 (3)	62.7 (2)	75.4 (3)
*Sas-6*	Ced	+	+	NA	9.5	31.9 (2)	36.7 (3)	19.6 (2)	79.1 (3)
*RpL32*		+	+	NA	101.6	100.0	100.0	100.0	100.0

These data prompted us to determine the transcription levels of *cid*, *Ndc80*, *Nnf1b*, *Mis12* and *Spc25*/*Mitch* in cells depleted of the NSL components. RT-qPCR showed that the transcripts of all these genes are substantially reduced in *Rcd1*, *Rcd5* and *MBD-R2* RNAi cells, but only weakly reduced in *wds* RNAi cells. In cells depleted of Rcd1, Rcd5 or MBD-R2, the *Nnf1b* and *Mis12* transcripts were below

30% of the control level, while the *cid* transcripts were approximately 50% of the control level; the *Spc25*/*Mitch* and *Ndc80* transcripts were roughly 70% of those of controls (Table 1). Importantly, the reductions of the transcript levels detected by RT-qPCR, although quantitatively lower, are absolutely proportional to those previously observed by microarray experiments (Table 1 and [12]). The transcription level of *Ndc80* detected by RT-qPCR does not match the strong reduction of the protein seen by Western blotting (Fig 3A). However, it is possible that this discrepancy is due to the downregulation of *Nuf2* transcription (Table 1), which might affect the quantity of the Ndc80 protein, as Nuf2 and Ndc80 are mutually dependent for their stability [34, 39].

We also determined whether RNAi against *Rcd1*, *Rcd5*, *MBD-R2* and *wds* affects the transcription of *asl*, *Sas-4* and *Sas-6* (Table 1). Sas-6, SAK and Ana2 form a conserved core module required for *Drosophila* centriole duplication [40, 41]. Consistent with the published ChIP data (Table 1), we found that in *Rcd1*, *Rcd5* and *MBD-R2* RNAi cells the *Sas-6* transcripts are substantially reduced compared with controls, ranging from 20% to 37% of the control level (Table 1). Reductions in the *asl* and *Sas-4* transcripts were less pronounced, ranging from 40–56% and 63–66% of the control levels, respectively. In *wds* RNAi cells the abundance of *Sas-4* and *Sas-6* was slightly reduced while the level of *asl* transcripts was not affected (Table 1). Thus, our direct measure of the transcript abundance determined by RT-qPCR is fully consistent with both the published datasets and with our hypothesis that both the centriole duplication and chromosome segregation phenotypes are caused by reduced transcription of specific mitotic gene sets.

## Discussion

### The mitotic defects elicited by depletion of the NSL components are mostly the consequence of impaired transcription of mitotic genes

The finding that Rcd1, Rcd5, MBD-R2 and Wds exhibit different localization patterns during mitosis, and yet cause very similar phenotypes when depleted raised the hypothesis that these proteins regulate mitosis by controlling the transcription of mitotic genes. This hypothesis is strongly supported by previous [12, 14] and current (see Table 1) findings indicating that Rcd1, Rcd5 or MBD-R2 depletion results in a substantial downregulation of several genes involved in centromere/kinetochore assembly and centriole duplication. This hypothesis is further corroborated by the observation that Wds depletion, which causes a limited reduction in mitotic gene transcription, also results in a milder mitotic phenotype compared to those caused by Rcd1, Rcd5 or MBD-R2 deficiency.

It has been previously shown that the NSL complex specifically binds the promoters of most housekeeping genes and activates a large subset of these genes. It has been further shown that subunits of the NSL complex co-localize at promoters of the target genes, and that the complex acts as a single functional unit [10, 14]. Consistent with these results, we found that RNAi-mediated silencing of *Rcd1*, *Rcd5* or *MBD-R2* results in identical defects in sister chromatid separation. These defects lead to PMLES, which have been previously observed in cells defective in MT-kinetochore interactions [17, 28, 32, 33]. Analysis of published datasets revealed that transcription of both *cid* and several kinetochore protein-coding genes is downregulated in Nsl1-depleted cells (Table 1; see [12]). Moreover, we showed that in *Rcd1*, *Rcd5* and *MBD-R2* RNAi cells the transcription levels of *cid*, *Mis12* and *Nnf1b*, and to lesser extent, those of *Ndc80* and *Spc25*/*Mitch*, are reduced with respect to controls. The same RNAi cells displayed fewer Cid signals and reduced levels of the Cid and Ndc80 proteins compared to control. Thus, these results collectively suggest that Rcd1, Rcd5 and MBD-R2 work together in interphase to regulate proper transcription of multiple genes encoding centromere and kinetochore components. We propose that reduced transcription of these genes disrupts proper kinetochore assembly, impairing kinetochore-MT interaction. There are at least two considerations that support this interpretation. First, there is a clear hierarchy in recruitment of the kinetochore proteins. Cid is required for recruitment of all kinetochore proteins; localization of the Mis12 complex (Mis12, Nnf1a, Nnf1b and Knm1) and Spc105R/KNL1 are interdependent, while recruitment of the Ndc80 complex (Ndc80, Nuf2, Spc25R/Mitch) requires both Spc105R/KNL1 and the Mis12 complex. Second, even components of the same complex such as Ndc80 and Nuf2 are mutually dependent for their stability/localization [34–36, 42]. These complex dependency relationships suggest that even relatively modest reductions in kinetochore proteins can generate synthetic effects leading to kinetochore dysfunction.

We have shown that Rcd1 and Rcd5 accumulate at the centrosomes while MBD-R2 localization is restricted to the chromosomes. Nonetheless, depletion of each of the three NSL components leads to a clear defect in centrosome duplication. Thus, it is unlikely that this centrosomal phenotype is caused by a direct effect of the NSL complex proteins on centrosome behavior. Here again, the most likely explanation is that defective centrosome duplication is due to reduced transcription of multiple genes required for proper centriole structure and duplication, such as *ana2*, *asl*, *SAK*, *Sas-4* and *Sas-6* [23, 40, 41, 43, 44]. The products of these genes also show dependencies in their recruitment at centrioles. For example, the SAK kinase recruits the centriole cartwheel components Sas-6 and Ana2 that are required for recruitment of Sas-4, which in turn recruits Asl [41, 44], suggesting that multiple, even modest, depletions of these proteins can impair the centriole duplication machinery. Thus, we propose that the centrosome duplication phenotype elicited by RNAi against *Rcd1*, *Rcd5* or *MBD-R2*, is due to reduced transcription of target genes required for centriole duplication.

Mitotic defects caused by depletion of transcription factors have been previously observed both in flies and mammals. For example, mutations in genes encoding subunits of the *Drosophila* TFIIH transcription complex have been previously shown to disrupt mitosis. The TFIIH holo-complex is comprised of two subcomplexes, a 7-proteins core complex (XPB, XPD, p62, p52, p44, p34 and p8) and the CAK (Cdk7, CycH and MAT1) complex [45, 46]. Mutations in *marionette* (*mrn*) that encodes *Drosophila* p52 cause defects in mitotic chromosome condensation and integrity in larval brains [47]. Recently it has been also shown that embryos produced by *Drosophila* females depleted of p8, p52, XPB or Cdk7 often exhibit mitotic defects; these defects include poorly condensed chromosomes, disorganized spindles, isolated centrosomes and chromosomes not associated with the spindle. These mitotic aberrations have been attributed to transcriptional downregulation of a set of critical genes. Specifically, embryos from *p8* mutants showed reduced transcription of 104 genes that encode factors involved in DNA replication or mitosis [48].

Another interesting example pointing to an involvement of transcription factors in mitotic regulation is provided by studies on the human MMXD complex, which includes the MIP18, MMS19 and XPD proteins; XPD is also a component of TFIIH complex and is responsible for Xeroderma pigmentosum (XP). *MMS19* or *MIP18* knockdown moderately reduces the XPD level but does not affect the levels of the other TFIIH subunits, suggesting that the MMXD complex can function independently of the TFIIH complex. MIP18, MMS19 and XPD localize to the mitotic spindle of human cells and their siRNA-mediated depletion results in monopolar and multipolar spindles; similar aberrant spindles were also observed in cells from XP patients [49]. However, the molecular mechanisms leading to this mitotic phenotype are not fully understood, and both a direct mitotic role of the MMXD complex and possible subtle defects in global transcription were considered [49]. Strong mitotic defects have also been observed in human cells upon siRNA-mediated inactivation of ERG, a transcription factor of the Erg family. However, in this case the mitotic defects have been attributed to failure to degrade the *Aurora A* and *B* mRNAs, and to a consequent excess of these mitotic kinases [50].

### Do the NSL components have direct mitotic roles?

While our data suggest that the chromosome segregation and the centrosome duplication phenotypes result from reduced transcription of mitotic genes during interphase, the localization of the NSL proteins to mitotic structures raises the possibility that these proteins might also play direct roles during cell division. A direct mitotic role of the KANSL complex components has been suggested in several studies on human cells. In HeLa cells, MCRS1 (Rcd5), KANSL1 (Nsl1/Wah) and KANSL3 (Rcd1/Nsl3) co-localize with the centrosomes at the center of the asters. KANSL1 and KANSL3 bind the MT minus ends, while MCRS1 does not directly bind MTs but is recruited at the minus ends by KANSL3 [7, 8]. RNAi-mediated silencing of the *MCRS1*, *KANSL1* or *KANSL3* resulted in very similar phenotypes consisting of misaligned chromosomes, a prolonged arrest in a prometaphase-like state, often followed by mitotic catastrophe [7, 8]. It has been suggested that MCRS1, KANSL1 and KANSL3 stabilize MTs, favor chromosome-driven MT formation, and promote correct kinetochore fiber dynamics [8]. WDR5, the human orthologue of Wds, has been also implicated in mitosis. WDR5 associates with the mitotic spindles [9] and is particularly enriched at the midbody dark zone [51]. Most interestingly, WDR5 depletion results in many cells that are highly reminiscent of the PMLES observed here. In most WDR5-depleted cells, the chromosomes did not properly align in the metaphase plate but are dispersed throughout unusually long anaphase-like spindles without undergoing sister chromatid separation, suggesting a defect in kinetochore-MT connection [9]. It has been also reported that RNAi-mediated knockdown of *WDR5* impairs completion of cytokinesis leading to multinucleated cells [51].

We have shown that MBD-R2 remains associated with the chromosomes throughout mitosis, just like some components of the TFIIH complex that localize to the chromosomes of dividing nuclei in *Drosophila* embryos [48]. However, we found that Rcd1 (KANSL3) and Rcd5 (MCRS1) are enriched at the centrosomes and at the midbodies, with Rcd5 accumulating in the midbody dark zone and Rcd1 excluded from this region but enriched at its sides. Finally, we showed that during mitosis Wds remains associated with a specific chromosome region that is likely to correspond to the nucleolus organizer; in addition, it accumulates at the centrosomes, the kinetochores and the midbody dark zone. These results raise the question of whether the accumulations of Rcd1, Rcd5 and Wds at the centrosomes/asters and the midbody reflect direct roles of these proteins during mitosis. It is unlikely that Rcd1, Rcd5 or Wds localization at the centrosomes is related to centrosome duplication, because defects in this process have been also observed in cells depleted of MBD-R2, which fails to accumulate at the centrosomes. It is also unlikely that these three proteins are required for aster formation, as in Rcd1-, Rcd5- or Wds-depleted cells these structures are morphologically normal. Postulating centrosomal functions for Rcd1, Rcd5 or Wds is extremely difficult because the centrosomes contain hundreds of proteins that do not appear to serve canonical centrosome functions, and it remains to be determined whether these proteins fulfill centrosome-related regulatory functions [52, 53]. Postulating direct roles of Rcd1, Rcd5 and Wds at the midbody is even more difficult. Their enrichment at the midbody suggests an involvement in cytokinesis. However, we did not notice any significant increase in the frequency of binucleated cells after *Rcd1*, *Rcd5* or *wds* RNAi, and none of these genes was detected in genome-wide RNAi-based screens aimed at identifying *Drosophila* genes required for cytokinesis [54, 55]. Moreover, proteomic analyses have shown that the midbody contains many proteins with no obvious roles in the execution of cytokinesis, and it is currently unknown whether they regulate some aspects of the process [56].

Studies on the human homologues of Rcd1, Rcd5 and Wds have shown that depletion of these proteins leads to chromosome segregation defects reminiscent of those observed in *Drosophila* cells [7, 8, 9]. These studies did not address the roles of Rcd1, Rcd5 and Wds on centrosome duplication and did not check whether their depletion leads to a reduced transcription of critical mitotic genes. However, it has been reported that KANSL3 (Rcd1) and MCRS1 (Rcd5) bind to MTs and physically interact with the TPX2 and MCAK spindle proteins, suggesting a direct participation of both KANSL components in the mitotic process [7, 8]. It has been also shown that WDR5 (Wds) interacts with the mitotic kinesin Kif2A and with the MLL complex that binds to MTs and regulates spindle assembly and chromosome segregation [9]. Although these findings do not exclude that downregulation of KANSL3, MCRS1 and WDR5 lowers the expression of a number of mitotic genes, they suggest that these KANSL subunits play direct mitotic roles. Likewise, we cannot exclude that Rcd1, Rcd5, MBD-R2 and Wds have some minor direct roles in chromosome segregation in *Drosophila* S2 cells. We have shown that Wds localizes to the kinetochores and it is possible that also small, cytologically undetectable, amounts of Rcd1, Rcd5, and MBD-R2 localize and function at kinetochores.

The human WDR5 protein localizes to dark zone of the midbody and is required for abscission during cytokinesis [51]. It is conceivable that Rcd1, Rcd5 and Wds play some functions in cytokinesis, and that these functions cannot be detected because they are masked by the chromosome segregation phenotype leading to PMLES. Alternatively, Rcd1, Rcd5 and Wds might play roles in cytokinesis that do not lead to a complete failure of the process; for example, they could regulate the timing of the events underlying cytokinesis either in its early or late stages, such as central spindle assembly and MT severing during abscission.

In conclusion, our data suggest that depletion of components of the NSL complex negatively affects centriole duplication and kinetochore assembly through downregulation of genes required for these processes. However, we have also shown that Rcd1, Rcd5 and Wds accumulate at the centrosomes and the midbody suggesting possible moonlighting functions for these proteins during mitosis. It is generally accepted that protein moonlighting occurred through the gradual transition from the original function to a novel function, an evolutionary process that involves the coexistence of two functions in the same protein. Because these functions should not be conflicting it has been also posited that evolution of moonlighting functions is favored when they are exerted in different cellular compartments [57, 58]. The components of the conserved NSL/KANSL complex are therefore in a very favorable situation to evolve secondary mitotic functions, as transcription is strongly reduced during cell division [59, 60]. In both *Drosophila* and human cells, the NSL/KANSL components appear to localize to mitotic structures. However, the localizations and functions of the NSL subunits are not identical to those of their KANSL counterparts. We would like to speculate that the components of the KANSL and NSL complexes are both evolving towards the acquisition of direct mitotic functions. However, while in human cells the functions of these proteins are integral to the mitotic process, in *Drosophila* they are not yet essential for mitosis. Although, further analyses are required to compare the mitotic functions of the KANSL and NSL complexes, the current results suggest their components are evolving secondary mitotic functions that are partially different.

## Materials and methods

### dsRNA production

All DNA templates for synthesis of dsRNAs specific to the CS or the UTRs of the *MBD-R2*, *Rcd1*, *Rcd5* and *wds* genes were amplified by PCR from a pool of cDNAs obtained from ovaries of 3-day-old wild-type females and from 0–2 hour wild-type embryos (for the primer sequences used, see S1 Table). The PCR products were purified using spin columns (BioSilica; http://biosilica.ru/). Synthesis of dsRNAs was done as described earlier [61], with the following minor modifications. Heating of the synthesized dsRNAs to 65°C and the subsequent slow cooling to room temperature were done before treatment with DNaseI; also, the phenol/chloroform extraction was omitted.

### RNAi treatments

S2 cells free from mycoplasma contamination were cultured in 39.4 g/L Shields and Sang M3 Insect medium (Sigma) supplemented with 0.5 g/L KHCO_3_ and 20% heat-inactivated fetal bovine serum (FBS) (Thermo Fisher Scientific) at 25°C. S2 cells expressing GFP-tagged proteins were cultured in 39.4 g/L Shields and Sang M3 Insect medium supplemented with 2.5 g/L bacto peptone (Difco), 1 g/L yeast extract (Difco) and 5% heat-inactivated FBS at 25°C. RNAi treatments were carried out as follows. 1×10^6^ cells were plated in 1 ml of serum-free medium in a well of a six-well culture dish (TPP) and 30 μg of CS dsRNA or 40 μg of UTR dsRNA (S1 Table) was added to each well. After a 1 hour incubation, 2 ml of the medium supplemented with 20% or 5% heat-inactivated FBS was added to each well and cells were grown for 3 days. After that, the second dose of the same dsRNA (30 μg of CS dsRNA or 40 μg of UTR dsRNA) was added to each sample and cells were grown for 2 additional days. In the case of the rescue experiments shown in S2 Table, together with the second dose of dsRNA, we added CuSO_4_ to the final concentration of 0.1 mM in the medium. Control S2 cell samples were prepared in the same way, but without addition of dsRNA.

### Reverse transcription and quantitative PCR

Gene-specific primers were designed by using Primer-BLAST (https://www.ncbi.nlm.nih.gov/tools/primer-blast/) or Primer3 (http://bioinfo.ut.ee/primer3-0.4.0/primer3/) software; primer sequences are provided in S4 Table. For each primer pair, the efficiency was determined by construction of a standard curve using dilutions of the cDNA prepared from S2 cells according to [62] (S4 Table). Total RNA was isolated from control and dsRNA-treated S2 cells using RNAzol RT reagent (MRC) according to the manufacturer’s instructions. Genomic DNA was eliminated using the RapidOut DNA Removal Kit (Thermo Fisher Scientific). Reverse transcription was performed with the RevertAid reverse transcriptase (Thermo Fisher Scientific) using 2 μg of total RNA in the presence of 2 U/μl of RNaseOut Recombinant RNase Inhibitor (Thermo Fisher Scientific). qPCR was carried out using BioMaster HS-qPCR SYBR Blue (2×) reagent kit (Biolabmix; http://biolabmix.ru/en/) and CFX96 Real-Time PCR Detection System (Bio-Rad). We used the following thermal cycling conditions: 5 minutes at 95°C, followed by 39 cycles of 15 seconds at 95°C, 30 seconds at 60°C, and 30 seconds at 72°C. Data were collected during each extension phase. Negative control templates (water and cDNA synthesized without reverse transcriptase) were included in each run. Measurements of gene expression were done at least in two biological replicates, each with three (or, in the case of the negative controls, in two) technical replicates. The relative mRNA quantification was determined using the ΔΔCq method. mRNA expression levels were normalized to those of the housekeeping gene *RpL32*.

### Generation of polyclonal antibody against Rcd1

A 488-aa portion of Rcd1 (corresponding to amino acids 133–620 of GenPept accession no. NP_610927.3) was expressed as GST-fusion in *E*.*coli* and subsequently purified as described in [63]. The purified GST-Rcd1 fusion protein was used to immunize mice. Immunization was performed at the Center for Genetic Resources of Laboratory Animals, Institute of Cytology and Genetics SB RAS. Polyclonal antibodies were affinity purified from serum as reported previously [63].

### Western blotting

S2 cells were collected by centrifugation and pellets were lysed in either RIPA buffer (Sigma) containing 1× Halt Protease and Phosphatase Inhibitor Cocktail (Thermo Fisher Scientific) or in lysis buffer (50 mM Hepes-KOH pH 7.6, 1 mM MgCl_2_, 1 mM EGTA, 1% Triton X-100, 45 mM NaF, 45 mM β-glycerophosphate, 0.2 mM Na_3_VO_4_) in the presence of a cocktail of protease inhibitors (Roche). Cell extracts were pelleted at 15,000*g* for 15 minutes at 4°C and the supernatants were analyzed by Western blotting. Lysates were run on an 8% or a 10% SDS-PAGE and transferred to an Amersham Protran Supported 0.45 μm Nitrocellulose Blotting Membrane (GE Healthcare) by wet or semi-dry transfer. Membranes were blocked for 30 minutes in 2% dry milk in PBT (PBS with 0.1% TritonX-100). Membranes were incubated overnight using following primary antibodies: mouse anti-Rcd1 (1:500, this study), rabbit anti-MBD-R2 (1:1000, Novus Biologicals 49940002), rabbit anti-Wds (1:1000, Novus Biologicals 40630002), rabbit anti-Ndc80 (1:1000; a gift of M. Goldberg, Cornell University), rabbit anti-Cid (1:500; Active Motif 39713), mouse anti-α-tubulin (1:5000, Sigma T6199), rabbit anti-beta-actin, (1:1000, Invitrogen PA5-16914), mouse anti-Lamin Dm0 (1:3500, Developmental Studies Hybridoma Bank ADL67.10) and mouse anti-β-actin-HRP-conjugated (1:5000, Santa Cruz Biotechnology sc-47778 HRP). The non-HRP-conjugated primary antibodies were detected with HRP-conjugated anti-mouse or anti-rabbit IgGs, using either the ECL detection kit (GE Healthcare) or the Novex ECL Chemiluminescent Substrate Reagent Kit (Thermo Fisher Scientific) following the manufacturer’s protocols.

### Generation of S2 cells expressing GFP-tagged proteins

Full-length CS of *MBD-R2* (nucleotides (NTs) 123–3629, GenBank accession no. NM_169461.3, but with 1433T>C, 1436G>C and 1893G>A NT substitutions), *Rcd1* (NTs 295–3492, GenBank accession no. NM_137083.4), *Rcd5* (NTs 101–1834, GenBank accession no. NM_139595.3, but with 748C>A NT substitution), *wds* (NTs 300–1382, GenBank accession no. NM_080245.5, but with 698T>C and 827C>T NT substitutions) and *asl* (NTs 98–3079, GenBank accession no. NM_141300.2, but with 376C>G, 2046A>G, 2872C>T and 3041A>G NT substitutions) were cloned in a piggyBac transposon-based plasmid vector upstream of and in frame with the enhanced GFP (for simplicity, referred to as GFP) CS. The plasmids also contained a blasticidin-resistance cassette and the sequence encoding mCherry-αTub84B (Cherry-tubulin) fluorescent fusion protein. The expression of all fluorescent fusion proteins is under the control of the copper-inducible *MtnA* promoter. S2 cells co-transfected with a plasmid encoding the fluorescent fusion proteins and a plasmid encoding piggyBac transposase were cultured in 39.4 g/L Shields and Sang M3 Insect medium supplemented with 2.5 g/L bacto peptone, 1 g/L yeast extract, 5% heat-inactivated FBS and 20 μg/ml blasticidin (Sigma) for two weeks at 25°C. The antibiotic was then removed from the culture medium. All cells were free from mycoplasma contamination. To induce expression of fluorescent fusion proteins, cells were grown in the presence of different CuSO_4_ concentrations (0.1, 0.25, 0.4 or 0.5 mM) for 12–14 hours before *in vivo* analysis or fixation.

### Immunofluorescence staining

All procedures were performed at room temperature. 2×10^6^ S2 cells were centrifuged at 800*g* for 5 minutes, washed in 2 ml of PBS (Sigma), and fixed for 10 minutes in 2 ml of 3.7% formaldehyde in PBS. Fixed cells were spun down by centrifugation, resuspended in 500 μl of PBS and placed onto a clean slide using Cytospin 4 cytocentrifuge (Thermo Fisher Scientific) at 900 rpm for 4 minutes. The slides were immersed in liquid nitrogen, washed in PBS, incubated in PBT (PBS with 0.1% TritonX-100) for 30 minutes and then in PBS containing 3% BSA for 30 minutes. The slides were then immunostained using the following primary antibodies, all diluted in PBT: mouse anti-α-tubulin (1:500, Sigma T6199), rabbit anti-Spd-2 (1:4,000, [21]), rabbit anti-Cid (1:300, Abcam ab10887), rabbit anti-CycB (1:100, [64]), and mouse anti-Rcd1 (1:50, this study). Primary antibodies were detected by incubation for 1 hour with goat FITC-conjugated anti-mouse IgG (1: 30, Sigma F8264) or goat Alexa Fluor 568-conjugated anti-rabbit IgG (1: 350, Invitrogen A11077).

In the attempt to stain GFP-tagged proteins with anti-GFP antibodies, cells expressing these proteins were collected as described above, and fixed (i) for 10 minutes in 2 ml of 3.7% formaldehyde in PBS or (ii) for 15 minutes in 4% paraformaldehyde in PBS, or (iii) for 10 minutes with 8% formaldehyde (methanol-containing) in PBS (Sigma). The slides obtained as described above were then stained with mouse anti-α-tubulin (1:500, Sigma T6199) and either with rabbit anti-GFP (1:200, Invitrogen A11122) or chicken anti-GFP (1:200, Invitrogen PA1-9533), which were detected by Alexa Fluor 568-conjugated goat anti-mouse IgG (1:300, Invitrogen A11031), Alexa Fluor 488-conjugated goat anti-rabbit IgG (1:300, Invitrogen A11034) or Alexa Fluor 488-conjugated goat anti-chicken IgG (1:300, Invitrogen A11039), respectively. These procedures stained the interphase nuclei but did not result in clear immunostaining of the spindle-associated GFP-tagged proteins.

All slides were mounted in Vectashield antifade mounting medium with DAPI (Vector Laboratories) to stain DNA and reduce fluorescence fading. Images were obtained on ZeissAxioImager.M2 using an oil immersion EC Plan-Neofluar 100x/1.30 lens (Carl Zeiss) and captured by 506 mono (D) High Performance camera (Carl Zeiss).

### Measurement of the spindle length

The spindle length in S2 cells was measured with the ZEN 2012 (Carl Zeiss) software, using the “Spline curve” tool and measure function. We considered only cells that did not appear to be polyploid with respect to the basic karyotype of S2 cells. To measure the spindle length in cells at different mitotic stages we drew a freehand line between the two poles along the spindle axis. The data obtained for each spindle type (prometaphase/metaphase; anaphase, telophase and PMLES) were compared using the Wilcoxon Signed-Rank Test and plotted using the BoxPlotR program (http://shiny.chemgrid.org/boxplotr/).

### Live cell imaging

Cells carrying a transgenic construct encoding Cherry-tubulin and either MBD-R2-GFP, Rcd1-GFP, Rcd5-GFP or Wds-GFP were grown for 12–14 hours in the presence of different concentrations of CuSO_4_ (0.1, 0.25, 0.4 or 0.5 mM). 500 μl aliquots of cell suspensions (5×10^5^ cells/ml) were then transferred to cell chambers (Invitrogen A-7816) containing coverslips treated with 0.25 mg/ml concanavalin A (Sigma-Aldrich C0412) placed on the bottom of the chambers. Observations were performed between 20 and 120 minutes after cell plating in the chamber at a Zeiss LSM 710 confocal microscope, using an oil immersion 100×/1.40 plan-apo lens and the ZEN 2012 software.

### Analysis of previously reported ChIP and RNA profiling datasets

To estimate the promoter binding by Rcd1/Nsl3 and MBD-R2, we first calculated the genome-wide distributions of these proteins. BAM files with Illumina sequencing reads obtained in ChIP-seq profiling of Rcd1 and MBD-R2 (and the corresponding Input) in S2 cells aligned to the *Drosophila melanogaster* genome release 5 (dm3) [14] were downloaded from ArrayExpress (https://www.ebi.ac.uk/arrayexpress/) (accession number: E-MTAB-1085). The data were transformed to the BED format using convert2bed in BEDOPS toolkit (version 2.4.35) (http://bedops.readthedocs.io/en/latest/index.html) [65] and genomic positions of aligned reads were converted to *Drosophila melanogaster* genome release 6 (dm6) using UCSC LiftOver tool (http://genome.ucsc.edu/cgi-bin/hgLiftOver). Next, we divided the genome (only sequences of chromosomes X, 2L, 2R, 3L, 3R and 4 were taken for the analysis) into bins of equal size (100 bp) and counted the number of reads in each bin. Then, we converted the counts in reads per million (RPM) values and calculated log_2_-transformed MBD-R2/Input and Rcd1/Input ChIP-seq ratios. Only bins with finite values were used for the further analysis (1,124,416 bins for log_2_(MBD-R2/Input) and 1,125,776 bins for log_2_(Rcd1/Input)).

To estimate the promoter binding by Nsl1 we first calculated the genome-wide distribution of the protein. Scaled log_2_(ChIP/Input) microarray-based data for two replicates of Nsl1 in S2 cells [12] were downloaded from GEO (https://www.ncbi.nlm.nih.gov/geo/) (accession number: GSE30991). Genomic positions of ChIP microarray probes were converted from *Drosophila melanogaster* genome release 4 (dm2) to 6 (dm6) using FlyBase *Drosophila* Sequence Coordinates Converter (http://flybase.org/convert/coordinates) and the log_2_(ChIP/Input) ratios from the replicates were averaged.

We next retrieved gene annotation data from Ensembl BioMart release 91 (http://www.ensembl.org/index.html). Promoters were arbitrary defined as gene regions spanning from -1000 to +101 bp relative to the transcription start sites (TSS). To measure the levels of Rcd1, MBD-R2 and Nsl1 binding to promoters, we identified all 100-bins (in the case of Rcd1 and MBD-R2) or microarray probes (in the case of Nsl1) that overlap with each promoter by 1 bp or more. Then, for each promoter, we averaged the log_2_ ChIP values of such bins or microarray probes. If there were more than one TSS for a gene, their log_2_ ChIP values were averaged as well. The exact values obtained are reported in S1 Table; in Table 1 we indicate with a “+” symbol all promoters that are enriched in Rcd1, MBD-R2 or Nsl1 ChIP samples compared to the rest of the genome (in nearly all cases, the promoter sequences analyzed are within the 5% of the most Rcd1-, MBD-R2- or Nsl1-enriched genomic sequences).

To measure the effects of Nsl1 deficiency on gene expression, normalized log_2_-transformed microarray-based data for gene expression in *GST*-RNAi (control; in three replicates) or *nsl1* RNAi (in two replicates) S2 cells [12] were downloaded from GEO (accession number: GSE30991) and the replicates were averaged. Next, we identified microarray probes that belong to each gene, averaged their values, and calculated the percentage of gene expression in Nsl1-depleted cells compared to control.

## Supporting information

S1 Fig Examples of mitotic figures in control, *Rcd1* RNAi, *Rcd5* RNAi and *MBD-R2* RNAi cells. Cells were stained for DNA (DAPI, blue) and α-tubulin (green). (A) metaphases; (B) PMLES with arched anaphase-like spindles; (C) PMLES with particularly elongated spindles; the cell on the bottom has decondensed chromosomes; (D) PMLES with telophase-like spindles. Scale bars, 5 μm.
(TIF)

S2 Fig Western blotting analysis of the levels of CuSO_4_-induced Rcd1-GFP, MBD-R2 GFP and Wds-GFP. (A) Representative Western blots showing that after induction with 0.1, 0.25 or 0.5 mM CuSO_4_, the ratios between the MBD-R2-GFP and endogenous MBD-R2 are 0.8, 1.7 and 2.0 (means from 3 different experiments), respectively. Ctr is a cell line that does not carry the *MBD-R2-GFP* construct, and RNAi is a Ctr line treated with *MBD-R2* dsRNA. Tubulin and Lamin Dm0 are loading controls. (B) Representative Western blots showing that after induction with 0.1, 0.25 or 0.5 mM CuSO_4_, the Rcd1-GFP levels are 1.4-, 2.5- and 3.9-fold (means from 3 different experiments) higher than that of endogenous Rcd1, respectively. Ctr is a cell line that does not carry the Rcd1-GFP construct, and RNAi is a Ctr line treated with *Rcd1* dsRNA. Tubulin is a loading control. A single asterisk designates an apparent Rcd1-GFP degradation band; two asterisks designate an aspecific band. (C) Representative Western blots showing that Wds-GFP expression after induction with 0.1, 0.25 or 0.5 mM CuSO_4_ is substantially higher than that of the endogenous protein (2.7-, 3.9- and 3.9-fold, respectively; means from 3 different experiments). Ctr is a cells line that does not carry the Wds-GFP construct, and RNAi is a Ctr line treated with *wds* dsRNA. Tubulin was used as a loading control. The asterisk designates an apparent degradation band, which is consistently observed in all experiments.
(TIF)

S3 Fig Formaldehyde-based fixation disrupts accumulation of some but not all GFP-tagged NSL subunits on mitotic structures. Cells were stained for DNA (DAPI, blue), and with anti-GFP (green) and anti-α-tubulin antibodies (red). (A) Localization patterns of the indicated GFP-tagged proteins in live cells (between brackets) and fixed cells (no brackets). +, protein accumulation;–absence of accumulation; +* enrichment limited to a specific chromosomal region (see panel E). Nucl., nucleus; Centros., centrosomes; Chrom., chromosomes; Kinetoch., kinetochores. (B) Examples of fixed Rcd1-GFP-expressing cells that fail to accumulate the tagged protein at the centrosomes and the midbody. (C) Examples of fixed Rcd5-GFP-expressing cells showing no centrosomal or midbody enrichments of the tagged protein. (D) Examples of fixed MBD-R2-GFP-expressing cells showing a clear association of the tagged protein with the chromosomes. (E) Examples of fixed Wds-GFP-expressing cells showing accumulations of the tagged protein in a discrete chromosomal region and in the dark zone of the midbody.
(TIF)

S1 Table dsRNAs used for RNA interference. (DOCX)

S2 Table Expression of GFP-tagged NSL proteins rescues the mitotic phenotypes elicited by RNAi-mediated depletion of their endogenous untagged counterparts. (DOCX)

S3 Table DNA sequences of mitotic gene promoters in S2 cells are enriched in Rcd1, MBD-R2 and Nsl1 in ChIP samples relative to other genomic DNA sequences. (DOCX)

S4 Table Primers used for RT-qPCR. (DOCX)

S1 Data Underlying data for Fig 1G. Spd-2 fluorescence intensity of prometaphase and metaphase centrosomes.
(XLSX)

S2 Data Underlying data for Fig 2D and 2E. Spindle lengths of prometaphases/metaphases, anaphases, telophases and PMLES in mock-treated, *Rcd1* RNAi, *Rcd5* RNAi, *MBD-R2* RNAi and *wds* RNAi cells. Prometa, prometaphases; Meta, metaphases; Ana, anaphases; Telo, telophases. In the RNAi cells, anaphases are very rare and were not taken into account.
(XLSX)

S3 Data Underlying data for Fig 3A. Band intensity quantification relative to the control and normalized to the loading controls (actin or tubulin) from 3 or more independent experiments carried out on *Rcd1*, *Rcd5*, *MBD-R2* and *wds* RNAi cells to evaluate the Cid and Ndc80 levels.
(XLSX)

S4 Data Underlying data for S2 Fig. Band intensity ratios between the indicated GFP-tagged proteins and their endogenous untagged counterparts detected with specific antibodies after induction with 0.1, 0.25, or 0.5 mM copper sulfate.
(XLSX)
