# Supplementary figures and images for: RNAi-mediated depletion of the NSL complex subunits leads to abnormal chromosome segregation and defective centrosome duplication in Drosophila mitosis
Source: PLoS Genet. 2019 Sep 17;15(9):e1008371. doi: 10.1371/journal.pgen.1008371 (PMC6772098; doi:10.1371/journal.pgen.1008371)

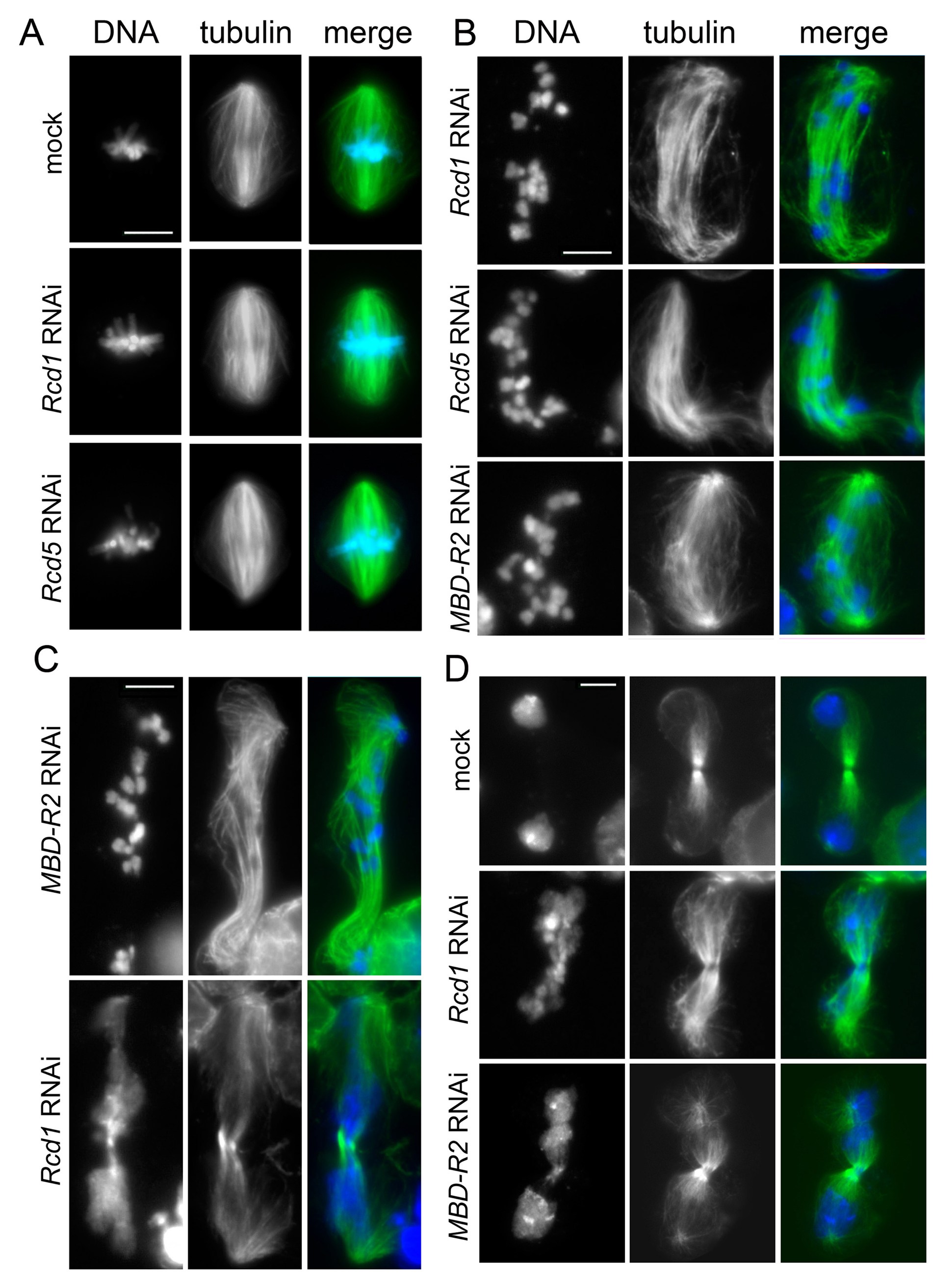

Supplement: S1 Fig — Cells were stained for DNA (DAPI, blue) and α-tubulin (green). (A) metaphases; (B) PMLES with arched anaphase-like spindles; (C) PMLES with particularly elongated spindles; the cell on the bottom has decondensed chromosomes; (D) PMLES with telophase-like spindles. Scale bars, 5 μm. (TIF) [file pgen.1008371.s001.tif]

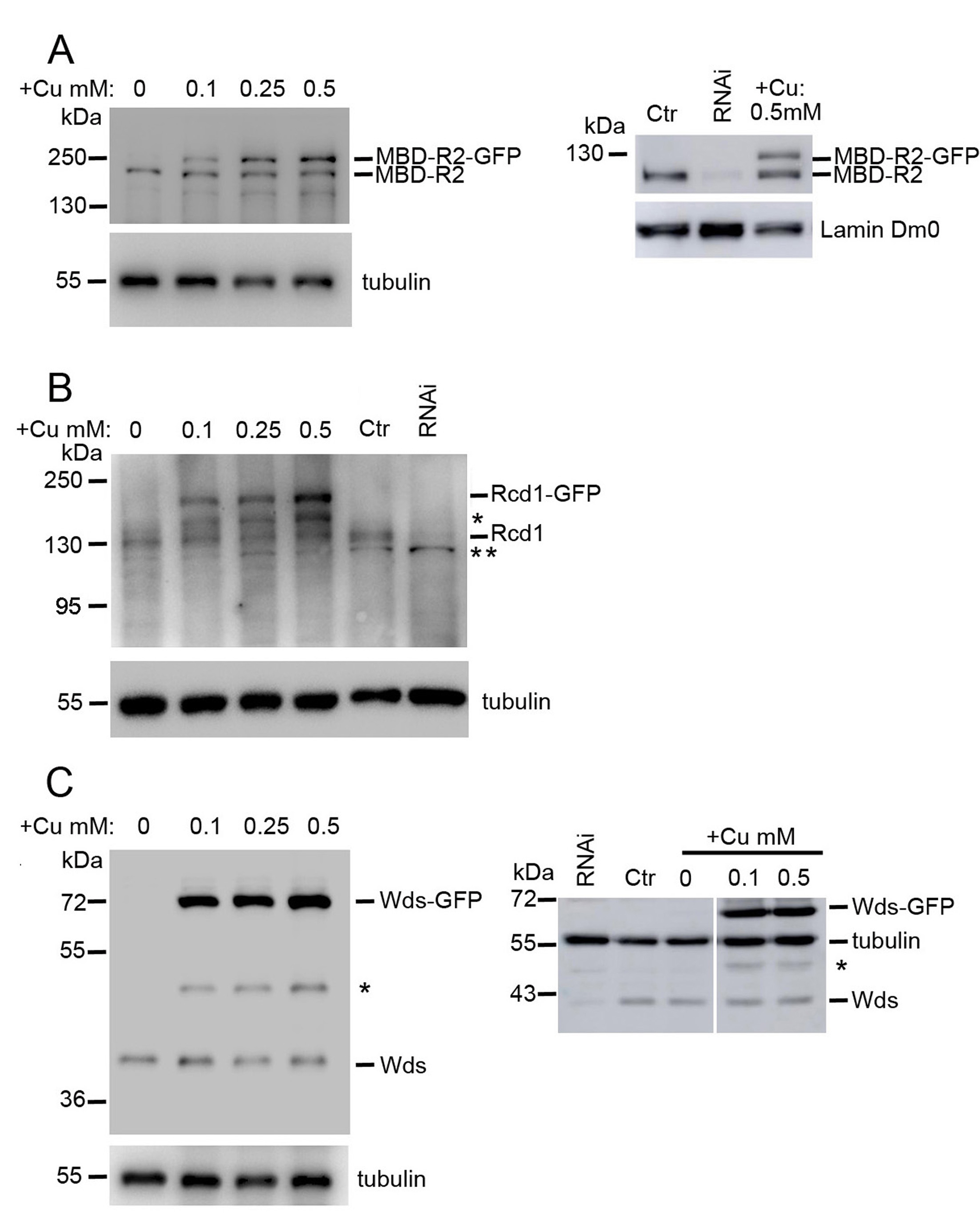

Supplement: S2 Fig — (A) Representative Western blots showing that after induction with 0.1, 0.25 or 0.5 mM CuSO4, the ratios between the MBD-R2-GFP and endogenous MBD-R2 are 0.8, 1.7 and 2.0 (means from 3 different experiments), respectively. Ctr is a cell line that does not carry the MBD-R2-GFP construct, and RNAi is a Ctr line treated with MBD-R2 dsRNA. Tubulin and Lamin Dm0 are loading controls. (B) Representative Western blots showing that after induction with 0.1, 0.25 or 0.5 mM CuSO4, the Rcd1-GFP levels are 1.4-, 2.5- and 3.9-fold (means from 3 different experiments) higher than that of endogenous Rcd1, respectively. Ctr is a cell line that does not carry the Rcd1-GFP construct, and RNAi is a Ctr line treated with Rcd1 dsRNA. Tubulin is a loading control. A single asterisk designates an apparent Rcd1-GFP degradation band; two asterisks designate an aspecific band. (C) Representative Western blots showing that Wds-GFP expression after induction with 0.1, 0.25 or 0.5 mM CuSO4 is substantially higher than that of the endogenous protein (2.7-, 3.9- and 3.9-fold, respectively; means from 3 different experiments). Ctr is a cells line that does not carry the Wds-GFP construct, and RNAi is a Ctr line treated with wds dsRNA. Tubulin was used as a loading control. The asterisk designates an apparent degradation band, which is consistently observed in all experiments. (TIF) [file pgen.1008371.s002.tif]

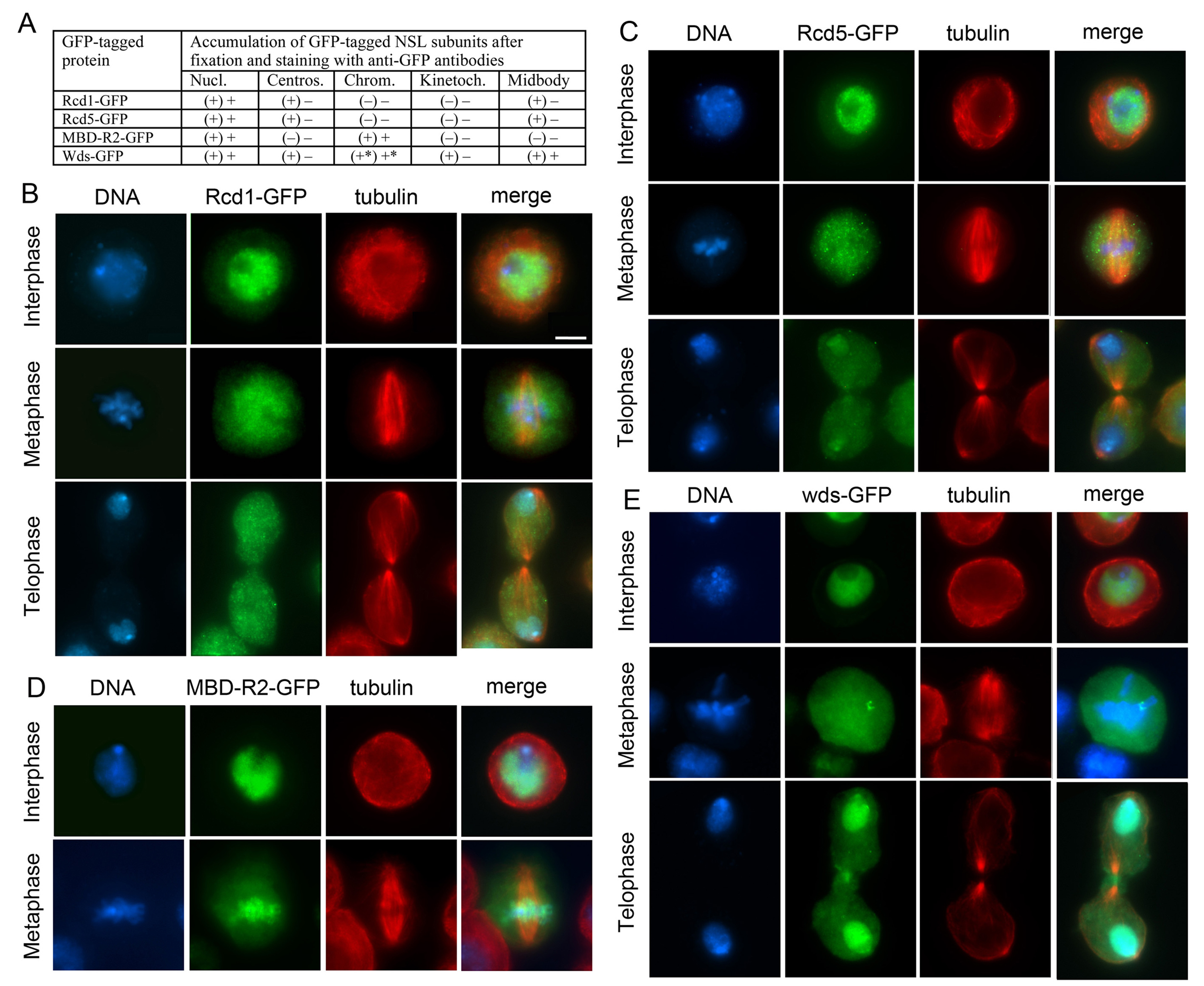

Supplement: S3 Fig — Cells were stained for DNA (DAPI, blue), and with anti-GFP (green) and anti-α-tubulin antibodies (red). (A) Localization patterns of the indicated GFP-tagged proteins in live cells (between brackets) and fixed cells (no brackets). +, protein accumulation;–absence of accumulation; +* enrichment limited to a specific chromosomal region (see panel E). Nucl., nucleus; Centros., centrosomes; Chrom., chromosomes; Kinetoch., kinetochores. (B) Examples of fixed Rcd1-GFP-expressing cells that fail to accumulate the tagged protein at the centrosomes and the midbody. (C) Examples of fixed Rcd5-GFP-expressing cells showing no centrosomal or midbody enrichments of the tagged protein. (D) Examples of fixed MBD-R2-GFP-expressing cells showing a clear association of the tagged protein with the chromosomes. (E) Examples of fixed Wds-GFP-expressing cells showing accumulations of the tagged protein in a discrete chromosomal region and in the dark zone of the midbody. (TIF) [file pgen.1008371.s003.tif]
