# Supplementary material for: RNAi-mediated depletion of the NSL complex subunits leads to abnormal chromosome segregation and defective centrosome duplication in Drosophila mitosis
Source: PLoS Genet. 2019 Sep 17;15(9):e1008371. doi: 10.1371/journal.pgen.1008371 (PMC6772098; doi:10.1371/journal.pgen.1008371)
Supplement: S1 Table — (DOCX) [file pgen.1008371.s004.docx]

**Table S1.** **dsRNAs used for RNA interference.**

| **dsRNA probe** | | | **Sequences of primers (5′->3′) used to amplify**  **DNA template(s) for dsRNA synthesis (2)** | **Notes** |
| --- | --- | --- | --- | --- |
| **name (1)** | **length,**  **bp** | **reference** |  |  |
| *MBD-R2-CS* | 937 | [17] | AGATCGAAGCAG  CCATGTCCAGCA |  |
| *Rcd1-CS* | 665 | [17] | AACGGGACAGAAAAAGTA  TAGCTCTTGGTGCC |  |
| *Rcd5-CS* | 530 | [16] | CTGCCAAATCCTTGCAAAACC  ATAGCAAAGGAGTGCCGT |  |
| *wds-CS* | 450 | This study | TGTGACCTTAAGCATTATTATTTTCAC  TTGGAGGCGCTGCTGTTCGAG |  |
| *MBD-R2-5UTR* | 122 | This study | ATTTATTGCGCTGTATTTTTCTGG  CGATGAAGTTCACCCAATTC | Equimolar mixture of  these two dsRNAs  was used for RNAi |
| *MBD-R2-3UTR* | 226 | This study | CATTTCTATTGATCACGGTTATAGGG  CATGAAATTATAATTTTCTGTATTCTCAC |  |
| *Rcd1-3UTR* | 175 | This study | AATTGGACAATGTTTTAATTGCGTG  TCGACTTTTTTTTATTTGTTCTTTATATGAAC |  |
| *Rcd5-3UTR* | 111 | This study | TTGCGATCAGAGCAGGCTGGC  ATGATTTATACATATGTCCATATGCTTTTTATTTC |  |
| *wds-5UTR* | 292 | This study | TATTAGTGTGACCTTAAGCATTATTATTTTC  ATGCGGATTCTGCTTATGCTC | Equimolar mixture of  these two dsRNAs  was used for RNAi |
| *wds-3UTR* | 213 | This study | TATCCAGCTGGAGGACGTTCG  TTTTGCCGTAGAATGTGAGGTTTAAAG |  |

(1) CS, coding sequence; 5UTR, 5′ untranslated region; 3UTR, 3′ untranslated region. (2) Each primer contained the following additional sequence of the T7 RNA polymerase binding site at the 5′ end (not shown in the table) (5′->3′): TAATACGACTCACTATAGGGAGG.
