# Supplementary material for: RNAi-mediated depletion of the NSL complex subunits leads to abnormal chromosome segregation and defective centrosome duplication in Drosophila mitosis
Source: PLoS Genet. 2019 Sep 17;15(9):e1008371. doi: 10.1371/journal.pgen.1008371 (PMC6772098; doi:10.1371/journal.pgen.1008371)
Supplement: S2 Table — (DOCX) [file pgen.1008371.s005.docx]

| **dsRNA used for RNAi in normal cells and in cells expressing the indicated GFP-tagged protein** | **# of cells**  **scored** | **Prometa (%)** | **Meta (%)** | **PMLES (%)** | **Ana (%)** | **Telo (%)** | **Telo LC (%)** |
| --- | --- | --- | --- | --- | --- | --- | --- |
| **Mock-treated normal cells** | 550 | 42.1 | 31.6 | 4.5 | 7.6 | 13.1 | 1.1 |
|  |  |  |  |  |  |  |  |
| ***UTR* *Rcd1* in normal cells** | 271 | 35.5 | 23.2 | 24.4* | 1.1 | 14.0 | 1.8 |
| **Mock in Rcd1-GFP** | 343 | 38.8 | 32.7 | 5.5 | 9.3 | 12.0 | 1.7 |
| ***UTR Rcd1* in Rcd1-GFP + Cu** | 344 | 42.7 | 26.5 | 3.8* | 8.7 | 16.6 | 1.7 |
| ***CS Rcd1* in Rcd1-GFP** | 109 | 34.9 | 15.6 | 41.3 | 1.8 | 4.6 | 1.8 |
|  |  |  |  |  |  |  |  |
| ***UTR Rcd5* in normal cells** | 204 | 26.6 | 29.4 | 23.0* | 3.9 | 14.2 | 2.9 |
| **Mock in Rcd5-GFP** | 338 | 42.8 | 34.9 | 2.1 | 8.3 | 9.2 | 2.7 |
| ***UTR Rcd5* in Rcd5-GFP +Cu** | 336 | 42.5 | 30.4 | 4.5* | 6.8 | 14.0 | 1.8 |
| ***CS Rcd5* in Rcd5-GFP** | 111 | 22.7 | 14.4 | 46.7 | 0.9 | 9.0 | 6.3 |
|  |  |  |  |  |  |  |  |
| ***UTR MBD-R2* in normal cells** | 321 | 35.2 | 19.9 | 26.2* | 1.9 | 13.1 | 3.7 |
| **Mock in MBD-R2-GFP** | 328 | 41.9 | 26.8 | 3.0 | 11.3 | 15.2 | 1.8 |
| ***UTR MBD-R2* in MBD-R2-GFP +Cu** | 339 | 36.0 | 32.1 | 4.7* | 8.0 | 16.5 | 2.7 |
| **CS *MBD-R2* in MBD-R2-GFP** | 179 | 29.1 | 15.6 | 41.9 | 1.1 | 7.3 | 5.0 |
|  |  |  |  |  |  |  |  |
| ***UTR wds* in normal cells** | 202 | 36.1 | 26.7 | 18.8* | 3.0 | 14.4 | 1.0 |
| **Mock in *wds-GFP*** | 338 | 44.3 | 30.2 | 2.7 | 6.8 | 15.1 | 0.9 |
| ***UTR* *wds* in Wds-GFP + Cu** | 385 | 35.3 | 28.6 | 7.0* | 7.0 | 19.2 | 2.9 |
| **CS *wds* in Wds-GFP** | 213 | 37.6 | 21.6 | 16.9 | 5.6 | 15.0 | 3.3 |

**Table S2. Expression of GFP-tagged NSL proteins rescues the mitotic phenotypes elicited by RNAi-mediated depletion of their endogenous untagged counterparts.** CS, coding sequence; UTR untranslated mRNA regions; Cu, copper sulfate (01 mM for 48 hours; see Materials and Methods). Prometa, prometaphases; Meta, metaphases; PMLES, prometaphase-like cells with elongated spindles; Ana, anaphases; Telo, telophases; Telo LC, telophases with lagging chromosomes. Note that after treatment with any given UTR dsRNA the frequencies of PMLES observed in normal cells are significantly higher than those seen in cells expressing the corresponding GFP-tagged transgene (see text for detailed explanation). In each rescue experiment, the compared values marked by asterisks are significantly different in the chi-square test with p < 0.001.
