# Supplementary material for: RNAi-mediated depletion of the NSL complex subunits leads to abnormal chromosome segregation and defective centrosome duplication in Drosophila mitosis
Source: PLoS Genet. 2019 Sep 17;15(9):e1008371. doi: 10.1371/journal.pgen.1008371 (PMC6772098; doi:10.1371/journal.pgen.1008371)
Supplement: S3 Table — (DOCX) [file pgen.1008371.s006.docx]

**Table S3.** DNA sequences of mitotic gene promoters in S2 cells are enriched in Rcd1, MBD-R2 and Nsl1 ChIP samples relative to other genomic DNA sequences. Data on Rcd1 and MBD-R2 are from [14]; data on Nsl1 are from [12].

| **Gene** | **Number of**  **promoters (TSSs)** | **ChIP binding to promoter(s), log_2_(NSL complex component/Input)**  **[percentile position in the genome-wide distribution*]** | | |
| --- | --- | --- | --- | --- |
|  |  | **Rcd1** | **MBD-R2** | **Nsl1** |
| *cid* | 1 | 2.902 [>99^th^] | 3.101 [ >99^th^] | NA |
| *Cenp-C* | 1 | 2.128 [ >97^th^] | 2.044 [ >97^th^] | NA |
| *Mis12* | 1 | 2.582 [ >98^th^] | 2.207 [ >98^th^] | NA |
| *Nnf1a* | 1 | 2.085 [ >97^th^] | 1.800 [ >97^th^] | NA |
| *Nnf1b* | 1 | 1.400 [ >96^th^] | 1.267 [ >94^th^] | 0.225 [ >77^th^] |
| *Kmn1* (*Nsl1*) | 1 | 1.518 [ >96^th^] | 1.589 [ >96^th^] | 1.182 [ >99^th^] |
| *Ndc80* | 1 | 1.366 [ >96^th^] | 1.780 [ >96^th^] | 0.326 [ >85^th^] |
| *Nuf2* | 1 | 2.426 [ >98^th^] | 2.117 [ >97^th^] | NA |
| *Spc25* (*Mitch*) | 1 | 1.280 [ >95^th^] | 0.823 [ >91^th^] | NA |
| *Spc105R (KNL1)* | 1 | 1.053 [ >94^th^] | 1.419 [ >95^th^] | NA |
| *Mad1* | 1 | 1.756 [ >97^th^] | 1.576 [ >96^th^] | 0.243 [ >78^th^] |
| *mad2* | 1 | 0.502 [>90^th^] | 0.337 [ >85^th^] | NA |
| *Bub1* | 2 | 1.795 [ >97^th^] | 1.659 [ >96^th^] | NA |
| *Bub3* | 1 | 1.677 [ >96^th^] | 1.801 [ >97^th^] | NA |
| *BubR1* | 1 | 1.032 [ >94^th^] | 0.909 [ >92^th^] | 0.425 [ >90^th^] |
| *Zw10* | 1 | 1.845 [ >97^th^] | 2.048 [ >97^th^] | 0.601 [ >95^th^] |
| *rod* | 1 | 2.502 [ >98^th^] | 2.367 [ >98^th^] | NA |
| *Zwilch* | 1 | 1.404 [ >96^th^] | 1.773 [ >96^th^] | NA |
| *cmet* | 1 | 2.091 [ >97^th^] | 1.693 [ >96^th^] | NA |
| *nudE* | 2 | 1.974 [ >97^th^] | 1.961 [ >97^th^] | 0.825 [ >98^th^] |
| *ana2* | 1 | 1.457 [ >96^th^] | 1.897 [ >97^th^] | 0.695 [ >97^th^] |
| *asl* | 1 | 1.377 [ >96^th^] | 1.209 [ >94^th^] | NA |
| *SAK* | 1 | 2.351 [ >98^th^] | 2.136 [ >97^th^] | NA |
| *Sas-4* | 1 | 2.363 [ >98^th^] | 2.612 [ >98^th^] | 0.805 [ >98^th^] |
| *Sas-6* | 1 | 1.521 [ >96^th^] | 1.921 [ >97^th^] | NA |
| *RpL32* | 3 | 2.501 [ >98^th^] | 2.557 [ >98^th^] | NA |

* 1,125,776 and 1,124,416 100-bp bins, and 384,636 microarray probes, all unambiguously assigned to chromosomes X, 2L, 2R, 3L, 3R and 4 and with finite log_2_ ChIP values, were analyzed for Rcd1, MBD-R2 and Nsl1, respectively. Note that the vast majority of promoter sequences analyzed are within the 5 % of the most Rcd1-, MBD-R2- or Nsl1-enriched genomic sequences. NA, data are not available.
