# Supplementary material for: RNAi-mediated depletion of the NSL complex subunits leads to abnormal chromosome segregation and defective centrosome duplication in Drosophila mitosis
Source: PLoS Genet. 2019 Sep 17;15(9):e1008371. doi: 10.1371/journal.pgen.1008371 (PMC6772098; doi:10.1371/journal.pgen.1008371)
Supplement: S4 Table — (DOCX) [file pgen.1008371.s007.docx]

**Table S4.** Primers used for RT-qPCR.

| **Target** | **Primer sequence (5′->3′)** | **Amplicon**  **size, bp** | **Primer**  **efficiency, %** |
| --- | --- | --- | --- |
| *asl* | ACGCCAGGTATAAGCCTCTTTC | 130 | 108.4 |
|  | TCACCATAATCCGTCCAAGCTC |  |  |
| *cid* | GAACAACTCAAAGTCGCCGAAC | 115 | 102.5 |
|  | GTTGTCCTGAAGCGTCAGTTG |  |  |
| *Mis12* | CTGCAGAGCGCGAACATATTG | 107 | 102.3 |
|  | CTTCTCGGCTTCCAGCAATTC |  |  |
| *Ndc80* | TCAAAGGTCAACGCGACTCA | 90 | 97.1 |
|  | ATCTAGCACGGGCTGAAAGG |  |  |
| *Nnf1b* | CACCGAAGCAGCCTTCAAAC | 123 | 104.5 |
|  | ATGGAGGCACACGACTCAAG |  |  |
| *Spc25* (*Mitch*) | CATGGAAAGCATTCACACGCTG | 90 | 102.9 |
|  | AGTACAGTGACACCCTTCACAC |  |  |
| *Sas-4* | AAGTGGAGGGACTGCAACTC | 120 | 112.2 |
|  | TCTCGTCACGGTAGTTTCGC |  |  |
| *Sas-6* | CCATGCGTAACTTTGCCCAG | 102 | 116.7 |
|  | ATCGCCGATTTTCTTTGCCC |  |  |
| *MBD-R2* | GGCCAAAAAGTGGGACGAAG | 139 | 101.5 |
|  | CAAACGGTACTGGCACTCCT |  |  |
| *Rcd1* | CCTCCAGATGATCAATACCGCACC | 109 | 93.9 |
|  | CAACTGCTTGAGTCCGGTTGCC |  |  |
| *Rcd5* | GCCAACAAAGCTCGACTCGGTC | 96 | 101.8 |
|  | GCGGATGGCGTTGATCAGCT |  |  |
| *wds* | TATCCGTCAAGCCCAACTACAC | 119 | 98.8 |
|  | TAGTTTATCAGCGGAGGAGCTG |  |  |
| *RpL32* | CTAAGCTGTCGCACAAATGG | 148 | 100.0 |
|  | AGGAACTTCTTGAATCCGGTG |  |  |
